# Dihydroartemisinin is potential therapeutics for treating late-stage CRC by targeting the elevated c-Myc level

**DOI:** 10.1038/s41419-021-04247-w

**Published:** 2021-11-05

**Authors:** Xianjing Hu, Sarwat Fatima, Minting Chen, Tao Huang, Yuen Wa Chen, Ruihong Gong, Hoi Leong Xavier Wong, Rongmin Yu, Liyan Song, Hiu Yee Kwan, Zhaoxiang Bian

**Affiliations:** 1 Centre for Cancer and Inflammation Research, School of Chinese Medicine, Hong Kong Baptist University, Hong Kong, China; 2 Biotechnological Institute of Chinese Materia Medical, Jinan University, Guangzhou, China; 3 Department of Pharmacology, College of Pharmacy, Jinan University, Guangzhou, China

**Keywords:** Target identification, Colorectal cancer

## Abstract

Currently, no frontline treatment is effective for the late-stage colorectal cancer (CRC). Understanding the molecular differences in different stages of CRC can help us to identify the critical therapeutic targets for designing therapeutic strategy. Our data show that c-Myc protein is highly expressed in late-stage CRC when compared with early-stage CRC in both clinical samples and in cell lines representing different cancer stages. Given that c-Myc is a well-known oncogenic driver in CRC, its high expression in the late-stage CRC may represent a critical therapeutic target for treating the cancer. Dihydroartemisinin treatment significantly increases c-Myc protein degradation and hence reduces its expression in CRC. The treatment also reduces CRC cell viability. Interestingly, dihydroartemisinin exhibits a more potent growth-inhibitory effect in late-stage CRC than the early-stage CRC. The treatment also possesses potent growth-inhibitory effects in mouse models bearing c-Myc-overexpressed CRC. The reduced c-Myc level and its reduced transcriptional activity reduce the expressions of acetyl-CoA carboxylase, fatty acid synthase, carnitine–palmitoyltransferase-1, and medium-chain acyl-CoA dehydrogenase in the cancer cells. Lipidomics study also shows that dihydroartemisinin treatment changes the metabolic phenotypes in CRC, reduces oxygen consumption, respiration, and ATP production, hence reduces the cell proliferation and induces apoptosis. Our study provides strong pharmacological evidence to support the translation of dihydroartemisinin for the treatment of late-stage CRC by targeting c-Myc.

## Introduction

Colorectal cancer (CRC) is one of the most common cancers worldwide. It is estimated that there will be 147,950 new CRC cases with 53,200 deaths in year 2020 [1]. The stages of CRC are directly associated with mortality. Currently, no frontline treatment is effective for the late-stage CRC, which results in high mortality. Understanding the molecular differences in different stages of CRC will help us to identify the critical therapeutic targets for the design of treatments.

Myc is a bHLH-zip pleiotropic transcription factor. The three isoforms c-Myc, l-Myc, and n-Myc have different expression patterns in cancers [2]. In CRC, c-Myc expression is dysregulated. Studies show that c-Myc expressions in CRC are elevated in 32–72% of the clinical cases [3–7]. These aberrant expressions of Myc are induced by the constitutive activation of mutated Ras [8], or via chromosomal translocation, gene amplification, and post-translational modifications. Since c-Myc regulates the transcription of the genes that are involved in cell proliferation, cell cycle, protein synthesis, cell migration, and adhesion [1, 2, 9, 10], its elevation drives the cancer to grow.

Furthermore, c-Myc is a master regulator of cancer metabolism that provides nucleic acids, proteins, and lipids for the cancer growth [11]. The aberrant c-myc expression in CRC is responsible for the cancer metabolic reprogramming, in which c-Myc induces 215 metabolic reactions by changing the expression levels of 121 metabolic genes and 39 transporter genes [12]. However, whether c-Myc expression is correlated with the CRC stages, which underlines the aggressiveness of the cancer growth at the late stage, is less studied.

Dihydroartemisinin is the active metabolite of all artemisinin compounds such as artemisinin, artesunate, and artemether. Dihydroartemisinin is available as a semisynthetic derivative of artemisinin in the market. A combination of artesunate–mefloquine has been used to treat malaria. Followed-up clinical studies show that dihydroartemisinin–piperaquine is a good alternative to artesunate–mefloquine because dihydroartemisinin–piperaquine is highly efficacious and is well tolerated by all age groups [13]. Dihydroartemisinin–piperaquine (Eurartesim) is an approved frontline therapy for the treatment of malaria [14, 15]. Its clinical application for other disease treatment is highly feasible.

In the current study, we aimed to investigate whether c-Myc levels were associated with CRC stages, and explored whether dihydroartemisinin targeted c-Myc in these CRC. Our data clearly showed that c-Myc expression increased as CRC stage advanced. By increasing the degradation of c-Myc protein and reducing energy production, dihydroartemisinin exhibited a high potency in inhibiting the growth of late-stage CRC.

## Results

### c-Myc expression is directly correlated with CRC staging

**Fig. 1:**
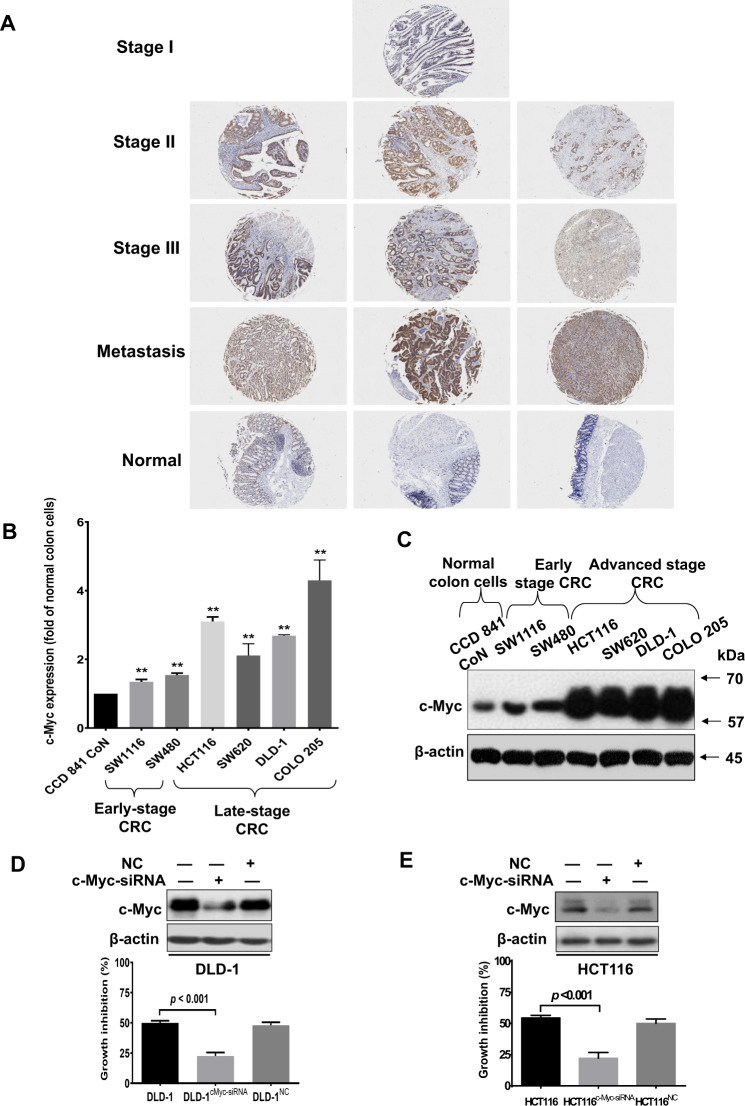
Expressions of c-Myc are higher in the late-stage than in the early-stage CRC. **A** Immunohistochemistry staining showing c-Myc expressions in clinical CRC samples at different stages of the development. **B** Real-time PCR showing the relative c-Myc mRNA levels, and (**C**) Western blot showing the c-Myc protein expressions in CRC cell lines derived from patients at different stages of the development. The inhibitory effect of dihydroartemisinin in c-Myc-knockout (**D**) DLD-1 (DLD-1^c-Myc-siRNA^) and (**E**) HCT116 (HCT116^c-Myc-siRNA^) cells. Nonspecific siRNA (NC) served as control. The data are shown as means ± SEM. *n* = 3 independent experiments, **p < 0.01 compared with CCD841 CoN.

We first examined whether c-Myc expression was associated with CRC stages. As shown in Fig. 1A, c-Myc expression was higher in CRC than in normal colon epithelial cells. More importantly, CRC at late stage had relatively higher c-Myc expressions when compared with the early stage, suggesting that c-Myc expression increases as CRC stage advances. To further investigate whether c-Myc expression is correlated with CRC staging, we also examined the protein-expression levels of c-Myc in different CRC cell lines that were derived from patients at different CRC stages. The SW1116 cells are of Dukes’ stage A, SW480 of Dukes’ stage B, SW620 and DLD-1 of Dukes’ stage C, and HCT116 and COLO205 are of Dukes’ stage D [16]. Our data clearly showed that CRC cell lines represent the advanced stage (HCT116, SW620, DLD-1, and COLO205), had higher c-Myc mRNA (Fig. 1B), and protein (Fig. 1C) expressions than those that represent the early stage (SW1116 and SW480), suggesting that both c-Myc mRNA and protein expressions increase as the CRC stage advances. These data strongly suggest that c-Myc expression is correlated with CRC staging. c-Myc is an oncogenic driver in CRC [2–7]. Indeed, our data also showed that knockdown of c-Myc significantly reduced the cell viability of DLD-1 (Fig. 1D) and HCT116 cells (Fig. 1E), suggesting that inhibiting c-Myc is a pragmatic therapeutic approach for treating late-stage CRC.

### Dihydroartemisinin treatment reduces c-Myc expression, and exhibits a potent growth-inhibitory effect in late-stage CRC

**Fig. 2:**
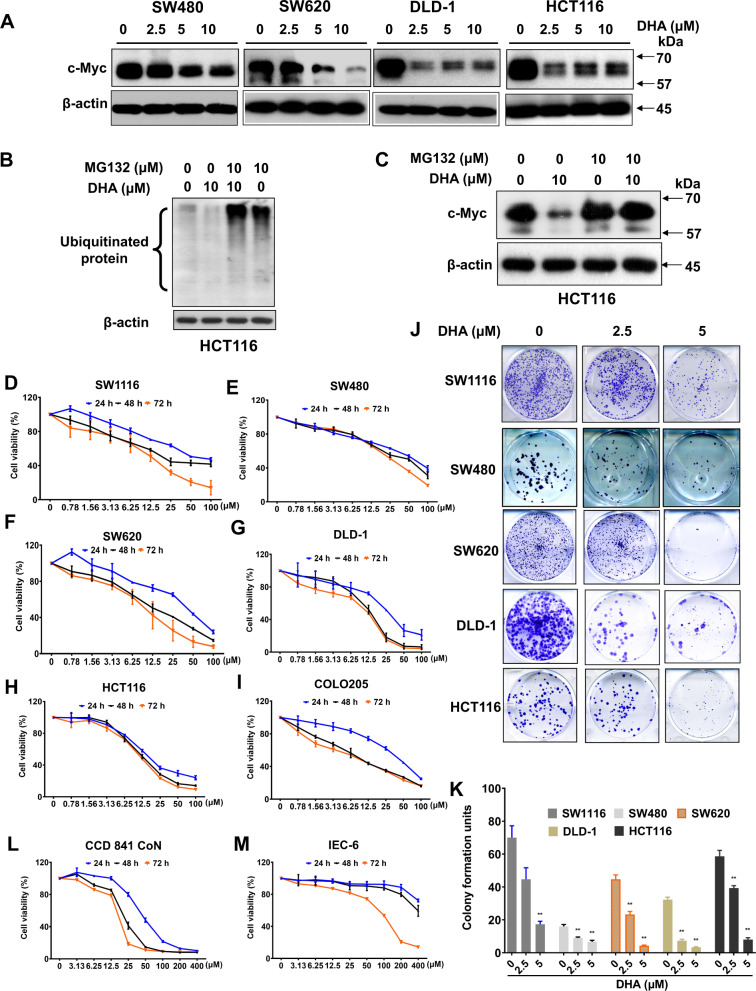
Dihydroartemisinin treatment reduces c-Myc expression and exhibits a potent growth inhibitory effect in late-stage CRC. **A** Expressions of c-Myc protein in CRC cells after dihydroartemisinin (DHA) treatment for 48 h at the indicated concentrations. **B** The ubiquitinated protein levels and (**C**) c-Myc protein levels in CRC cells in the presence or absence of MG132 upon dihydroartemisinin (DHA) challenge at the indicated concentrations. The cell viability of (**D–I**) CRC cells, (**L**) human normal colon epithelial cells, and (**M**) rat epithelial cells after dihydroartemisinin (DHA) treatments at the indicated concentrations. (**J**) The colony formation of CRC cells after dihydroartemisinin (DHA) treatments at the indicated concentrations and (**K**) the quantification of the colony formation. The data are shown as means ± SEM. *n* = 3 independent experiments, **p < 0.01 compared with control.

Next, we aimed to screen for a compound that was effective in treating late-stage CRC by targeting c-Myc (Supplementary Fig. S1). After a high-throughput screening, we found that dihydroartemisinin treatment significantly reduced c-Myc protein levels in SW480, and the late-stage CRC such as SW620, DLD-1, and HCT116 in a dose-dependent manner (Fig. 2A). However, the treatment did not affect c-Myc mRNA level (Supplementary Fig. S2A–D), suggesting that dihydroartemisinin regulates c-Myc expression at the post-transcriptional level. Interestingly, in the presence of MG132, which is a potent inhibitor of the proteolytic activity of the 26 S proteasome complex, dihydroartemisinin treatment significantly increased the ubiquitinated protein level (Fig. 2B) and the c-Myc protein level (Fig. 2C). Our data suggest that dihydroartemisinin increases the c-Myc protein degradation in the CRC cells. Reduced c-Myc levels will reduce the cancer growth. Indeed, dihydroartemisinin treatments significantly reduced the growth of SW1116 (Fig. 2D), SW480 (Fig. 2E), as well as the late-stage CRC such as SW620 (Fig. 2F), DLD-1 (Fig. 2G), HCT116 (Fig. 2H), and COLO205 (Fig. 2I). The growth inhibition was also suggested by the reduced colony formation (Fig. 2J, K). Interestingly, at the same dihydroartemisinin concentration, the growth inhibition was more prominent in the late-stage CRC (Fig. 2J), suggesting that dihydroartemisinin is more effective in inhibiting the growth of the late-stage CRC. Indeed, the calculated IC_50_ of dihydroartemisinin for the early-stage CRC represented by SW1116 and SW480, were 63.79 ± 9.57 µM and 65.19 ± 5.89 µM, respectively (Table 1). The IC_50_ of dihydroartemisinin for late-stage CRC represented by SW620, DLD-1, HCT116, and COLO205 was much lower, ranging from 15.08 ± 1.70 µM to 38.46 ± 4.15 µM after 24-h treatment (Table 1). The treatment also reduced the growth of normal human colon epithelial cells (CCD841 CoN) (Fig. 2L) and rat small-intestine epithelial cells (IEC-6) (Fig. 2M), but with higher IC_50_ values compared with the late-stage CRC (Table 1). Our in vitro data strongly suggest that dihydroartemisinin is potential therapeutics for treating late-stage CRC by targeting c-Myc.

**Table 1:** IC_50_ of dihydroartemisinin in different cell lines.

Cell types	Cell lines	IC_50_ (µM)		
		24 h	48 h	72 h
Human normal colon epithelial cells	CCDc 841 CoN	53.51 ± 2.33	29.14 ± 0.42	21.96 ± 0.31
Normal rat small intestine epithelial cells	IEC-6	>400	>400	81.77 ± 3.01
Dukes’ type A	SW1116	63.79 ± 9.57	39.80 ± 1.22	23.28 ± 0.63
Dukes’ type B	SW480	65.19 ± 5.89	28.92 ± 7.54	10.43 ± 2.73
Dukes’ type C	SW620	35.96 ± 8.76	14.03 ± 2.24	10.59 ± 1.68
Dukes’ type C	DLD-1	15.08 ± 1.70	10.65 ± 0.69	7.51 ± 0.69
Dukes’ type D	HCT116	19.53 ± 1.24	13.97 ± 1.31	11.33 ± 0.63
Dukes’ type D	COLO 205	38.46 ± 4.15	9.63 ± 1.12	7.55 ± 1.15

The data are shown as the means ± SEM. *n* = 3 independent experiments.

### Dihydroartemisinin treatments possess potent growth-inhibitory effects in mouse models bearing c-Myc-overexpressed CRC

**Fig. 3:**
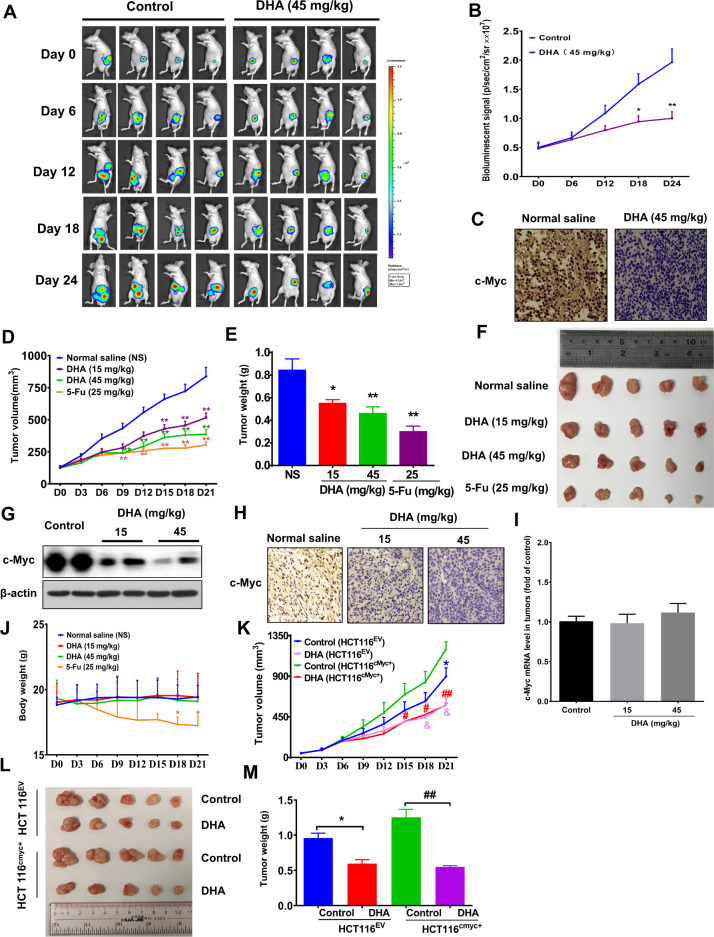
Dihydroartemisinin treatments possess potent growth-inhibitory effects in mouse models bearing c-Myc-overexpressed CRC. **A** Bioluminescence signal of the tumors in the HCT116-Fluc-Neo/GFP-puro cell-bearing orthotopic mouse models after dihydroartemisinin (DHA) treatment and (**B**) quantification of the signal. The data are shown as means ± SEM. *n* = 4 mice in each group. *p < 0.05, **p < 0.01 compared with control. **C** Immunohistochemistry staining showing c-Myc expression in the tumor tissues after the treatment. **D** Tumor volume, (**E, F**) tumor weight of the DLD-1-bearing xenograft mouse model after dihydroartemisinin (DHA) treatments. **G** Western blot and (**H**) immunohistochemistry staining showing c-Myc protein expressions, and (**I**) c-Myc mRNA levels in the tumor of the DLD-1-bearing xenograft mouse models after the treatments. **J** Body weight of the DLD-1-bearing xenograft mouse models after the treatment. The data are shown as means ± SEM. *n* = 5 mice in each group. *p < 0.05, **p < 0.01 compared with normal saline group. **K** Tumor volume, (**L**) tumor size and (**M**) tumor weight of the xenograft mouse models bearing HCT116^cmyc+^ or HCT116^EV^ cells after dihydroartemisinin (DHA) treatment. The data are shown as means ± SEM. *n* = 5 mice in each group. *p < 0.05 compared with control in HCT116^EV^-bearing mouse model. ^#^*p* < 0.05, ^#^*p* < 0.01, ^###^*p* < 0.001 compared with control in HCT116^cmyc+^-bearing mouse model. HCT116^cmyc+^, c-Myc-overexpressed HCT116; HCT116^EV^, empty vector-transfected HCT116.

We also examined the growth-inhibitory effect of dihydroartemisinin in CRC by establishing different mouse models. We first established orthotopic CRC mouse models by inoculating the mice with HCT116 cells that represent the late-stage CRC. These HCT116 cells are stably overexpressed GFP protein (HCT116-Fluc-Neo/GFP-puro) for in vivo tracking of the tumor growth. Dihydroartemisinin has been approved for treating malaria, based on the European Medicines Agency, the dose ranges from 1.67 to 3.64 mg/kg/day, while the target dose of dihydroartemisinin is 4 mg/kg/day with reference to the WHO guidelines. To explore the effect of dihydroartemisinin in treating CRC in mouse models, we converted these human dosages that are safe for human-to-mouse dosages. The converted mouse dose range of dihydroartemisinin is 20.54–49.20 mg/kg/day. In our in vivo studies, as shown in Fig. 3A, B, dihydroartemisinin treatments at 15 mg/kg or 45 mg/kg significantly reduced tumor growth in these mice starting from day 18 of the treatment. The treatment also significantly reduced c-Myc protein expressions in the tumors in these mice (Fig. 3C). To further suggest the potent growth-inhibitory effect of dihydroartemisinin in vivo, we next established xenograft mouse model by subcutaneously inoculated DLD-1 cells into the nude mice. DLD-1 cells also represent the late-stage CRC that has high c-Myc expression (Fig. 1C). 5-Fu served as positive control. Our data showed that the dihydroartemisinin treatments significantly reduced the tumor volume (Fig. 3D) and tumor weight (Fig. 3E, F) in these mice. The treatment also significantly reduced the protein expressions of c-Myc in the tumors as demonstrated by Western blot analysis (Fig. 3G) and immunohistochemistry staining (Fig. 3H). However, the treatment did not affect the c-Myc mRNA level (Fig. 3I), which was consistent with the in vitro findings (Supplementary Fig S2). Although 5-Fu also reduced the tumor volume and tumor weight (Fig. 3D–F), it also significantly reduced the body weight of the mice (Fig. 3J), implying that 5-Fu may have toxicity in the mice and dihydroartemisinin is preferable therapeutics with potent growth-inhibiting effect and no obvious toxicity. Although the late-stage CRC cells (HCT116 and DLD-1) we used for establishing these mouse models express higher c-Myc protein levels than those that represent the early stage, we tried to further increase the c-Myc expression in these cells and examined whether it would affect the potency of dihydroartemisinin in CRC. We overexpressed c-Myc in the HCT116 cells (HCT116^cMyc+^), empty vector transfection served as control (HCT116^EV^). Fig. 3K–M clearly shows that HCT116^cMyc+^ was grown into a bigger tumor than HCT116^EV^, suggesting the oncogenic role of c-Myc in CRC in our mouse models. Dihydroartemisinin treatment significantly reduced the cancer growth in these mice-bearing HCT116^cMyc+^ (Fig. 3K–M). Taken together, our in vivo data strongly suggest that dihydroartemisinin is potential therapeutics for treating late-stage CRC by reducing the c-Myc expression.

### Dihydroartemisinin reduces c-Myc transcriptional activity and its downstream targets involved in lipid metabolism

**Fig. 4:**
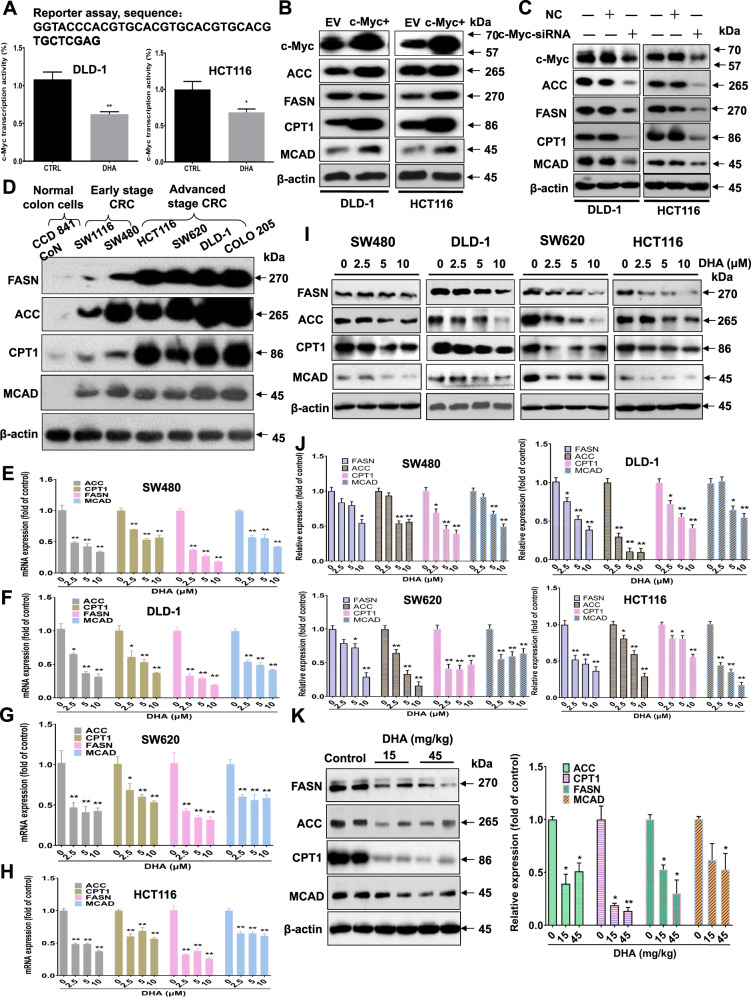
Dihydroartemisinin reduces c-Myc transcriptional activity, and its downstream targets involved in lipid metabolism. **A** c-Myc reporter activity in DLD-1 and HCT116 cells after dihydroartemisinin (DHA, 10 µM) treatment. The data are shown as means ± SEM, *n* = 3 independent experiments, *p < 0.05, **p < 0.01 compared with control. **B** Western blot showing the expressions of acetyl-CoA carboxylase (ACC), fatty acid synthase (FASN), carnitine palmitoyltransferase I (CPT-1), and medium-chain acyl-CoA dehydrogenase (MCAD) in c-Myc-overexpressed (c-Myc^+^) CRC cells, empty-vector (EV) transfection served as control. **C** Western blot showing the expressions of ACC, FASN, CPT-1, and MCAD in the CRC cells with c-Myc knockdown by siRNA. Nonspecific siRNA (NC) served as control. **D** Western blot showing the expressions of ACC, FASN, CPT-1, and MCAD in the CRC cell lines representing different CRC stages. The mRNA expressions of ACC, FASN, CPT-1, and MCAD in (**E**) SW480, (**F**) DLD-1, (**G**) SW620, and (**H**) HCT116 cells after dihydroartemisinin (DHA) treatments for 48 hr at the indicated concentrations. **I** Western blot and (**J**) the quantification of the expressions of ACC, FASN, CPT-1, and MCAD in different CRC cell lines after dihydroartemisinin (DHA) treatments at the indicated concentrations. The data are shown as means ± SEM, *n* = 3 independent experiments, *p < 0.05, **p < 0.01 compared with control. **K** Western blot and the quantification showing the protein expressions of ACC, FASN, CPT-1, and MCAD in the xenograft tissues of the DLD-1-bearing xenograft mouse model after dihydroartemisinin (DHA) treatments at the indicated doses. The data are shown as means ± SEM, *n* = 5 mice in each group, *p < 0.05, **p < 0.01 compared with control.

We next investigated the mechanism of action underlying the growth-inhibitory effects of dihydroartemisinin in CRC. Our data showed that dihydroartemisinin reduced c-Myc transcriptional activity in CRC cells as demonstrated in the c-Myc reporter assay (Fig. 4A). c-Myc regulates the expressions of the genes that are involved in the metabolic pathways [16]. In c-Myc-overexpressed DLD-1 and HCT116 cells, expressions of acetyl-CoA carboxylase (ACC), fatty acid synthase (FASN), carnitine palmitoyltransferase-1 (CPT-1), and medium-chain acyl-CoA dehydrogenase (MCAD) were significantly increased (Fig. 4B); whereas knockdown of c-Myc in these cells reduced the expressions of ACC, FASN, CPT-1, and MCAD (Fig. 4C). These data suggest that high level of c-Myc increases the expressions of lipogenic and beta-oxidation enzymes in CRC. Indeed, in late-stage CRC that expresses high endogenous c-Myc levels, the expressions of ACC, FASN, CPT-1, and MCAD were also higher than those of the early-stage CRC (Fig. 4D). More importantly, our data showed that dihydroartemisinin treatments significantly reduced the mRNA levels (Fig. 4E–H) and protein expressions (Fig. 4I, J) of ACC, FASN, CPT-1, and MCAD in these CRC cells, and in the tumors of the CRC-bearing xenograft mouse models (Fig. 4K).

### Dihydroartemisinin changes the metabolic phenotypes in CRC, regulates the c-Myc-mediated lipid metabolism, and reduces energy production that inhibits cancer growth

**Fig. 5:**
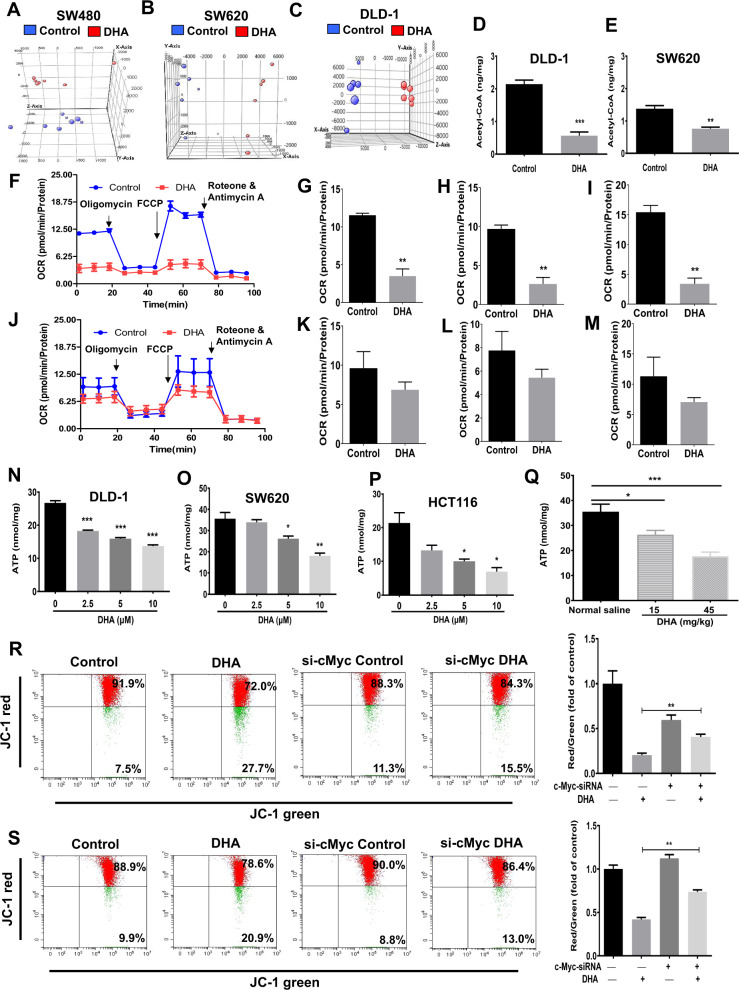
Dihydroartemisinin changes the metabolic phenotypes in CRC, regulates the c-Myc-mediated lipid metabolism, and reduces energy production that inhibits cancer growth. Principal component analysis of the lipid samples in (**A**) SW480 cells, (**B**) SW620 cells, and (**C**) DLD-1 cells after dihydroartemisinin (DHA) treatments. Acetyl-CoA levels in (**D**) DLD-1 and (**E**) SW620 cells after dihydroartemisinin (DHA, 10 µM) treatments for 48 hr. **F** The oxygen-consumption rate (OCR) and (**G**) quantification of OCR, (**H**) basal respiration, and (**I**) maximal respiration of the HCT116 cells upon dihydroartemisinin (DHA, 10 µM) challenge, vehicle served as control. **J** The oxygen-consumption rate (OCR) and (**K**) quantification of OCR, (**L**) basal respiration, and (**M**) maximal respiration of the c-Myc-knockdown HCT116 (HCT116^c-Myc-siRNA^) cells upon dihydroartemisinin (DHA, 10 µM) challenge, vehicle served as control. ATP levels in (**N**) DLD-1, (**O**) SW620, (**P**) HCT116 cells, and in (**Q**) the xenograft tissues of the DLD-1-bearing xenograft mouse models after dihydroartemisinin (DHA) treatments at the indicated concentrations or doses. Measurements of mitochondrial membrane potential with JC-1 dye in (**R**) HCT116 or c-Myc-knockdown HCT116 (HCT116^cMyc-siRNA^) cells and in (**S**) DLD-1 cells or c-Myc-knockdown DLD-1 (DLD^cMyc-siRNA^) cells after dihydroartemisinin (DHA, 10 µM) treatments. The data are shown as means ± SEM. *n* = 3 independent experiments or *n* = 5 mice in each group, *p < 0.05, **p < 0.01, ***p < 0.001 as indicated.

Since dihydroartemisinin treatment reduces the lipogenic and beta-oxidation enzyme expressions, we examined the effects of the treatments on lipid metabolism in CRC cells with LC/MS lipidomics. As shown in Fig. 5A–C, samples from the treatment group and vehicle group were clustered by three principal components in the principal component analysis (PCA), suggesting that dihydroartemisinin changes the lipid profiles in these cells. The alternations of the lipid species were illustrated in the heat maps and hierarchical clustering (Supplementary Fig S3A–C) and the correlation analysis of differential metabolites in these samples was shown in Supplementary Fig S3D–F. Our data also showed that dihydroartemisinin treatment reduced the total fatty acid levels in the CRC cells (Supplementary Fig S4A–C). For example, the levels of the immediate end product of de novo fatty acid synthesis, palmitic acid (PA) (C16:0), its elongation product stearic acid (C18:0), and the subsequent desaturation product oleic acid (C18:1) in the CRC cells were significantly reduced after the treatment (Table 2). The reduced PA levels are further suggested by the reduced levels of C16:0-incorporated glycerol lipids (Supplementary Fig S4D–F) and C16:0-incorporated phospholipids (Supplementary Fig 4G–I) in these cells. Data in the lipidomics study strongly suggest that dihydroartemisinin modulates the c-Myc-mediated fatty acid metabolism and inhibits de novo fatty acid synthesis in the CRC cells.

**Table 2:** Quantification of palmitic acid (PA), stearic acid (SA) and oleic acid (OA) in SW480, SW620 and DLD-1 cells after dihydroartemisinin treatments.

Cell lines	Concertation (μg/g protein)	Control	Dihydroartemisinin
SW480	PA	0.3403 ± 0.1905	0.1870 ± 0.0594*
	SA	0.0542 ± 0.0198	0.0350 ± 0.0046*
	OA	0.0453 ± 0.0267	0.0306 ± 0.0104
SW620	PA	1.2081 ± 0.1656	0.7720 ± 0.1433***
	SA	0.2634 ± 0.0396	0.1436 ± 0.0643**
	OA	0.0313 ± 0.0053	0.0138 ± 0.0051***
DLD-1	PA	1.2862 ± 0.2796	0.5294 ± 0.2004***
	SA	0.3249 ± 0.1025	0.1252 ± 0.0528***
	OA	0.0362 ± 0.0025	0.0088 ± 0.0049***

The data are shown as the means ± SEM. *n* = 3 independent experiments, *p < 0.05, *p < 0.01, ***p < 0.001 compared to control.

Since dihydroartemisinin treatment also reduced the expressions of CPT1 and MCAD, we next examined whether the treatments would affect the metabolic phenotypes in the cells. The abundance of acetyl-CoA reflects the energetic state of the cell [17]. Our data showed that dihydroartemisinin treatments significantly reduced the expression of acetyl-CoA in DLD-1 (Fig. 5D) and SW620 cells (Fig. 5E), suggesting that dihydroartemisinin reduces the energy state in the cells. We also used Bioscience XF Analyzers to monitor the oxygen-consumption rates (OCR) of the living CRC cells upon dihydroartemisinin challenge. Our data clearly showed that dihydroartemisinin significantly reduced OCR (Fig. 5F, G), basal respiration (Fig. 5H), and maximal respiration (Fig. 5I) in the HCT116 cells and in DLD-1 cells (Supplementary Fig. S5A–D); these inhibitory effects were abolished when c-Myc was knocked down in these cells (Fig. 5J,M and Supplementary Fig. S5E–H). Furthermore, dihydroartemisinin treatment reduced ATP production in these cells (Fig. 5N–P). In the DLD-1-bearing xenograft mouse model, we also found that dihydroartemisinin reduced the ATP levels in the xenograft tissues (Fig. 5Q). It is known that mitochondria-membrane potential is required for ATP production. Since the treatments reduced ATP production, we expected that the mitochondria-membrane potential in these cells was also affected. Indeed, the dihydroartemisinin treatments led to the collapse of the mitochondrial-membrane potential in HCT116 (Fig. 5R) and DLD-1 (Fig. 5S) cells, which was ameliorated after c-Myc was knocked down in these cells (Fig. 5R, S). Our data strongly suggest that dihydroartemisinin reduces OCR and leads to a collapse in mitochondria-membrane potential in a c-Myc-dependent manner in CRC.

OCR indicates the mitochondrial oxidative phosphorylation, while extracellular acidification rate (ECAR) measures the glycolysis in the cells. Glycolysis also provides substrates for mitochondrial oxidative phosphorylation for generating ATP. Therefore, we investigated whether dihydroartemisinin would also affect ECAR in CRC. Our data showed that dihydroartemisinin did not significantly affect ECAR, the glycolysis, glycolytic capacity, and the glycolytic reserve in HCT116 (Supplementary Fig S6A, B) and DLD-1 cells (Supplementary Fig S6C, D).

**Fig. 6:**
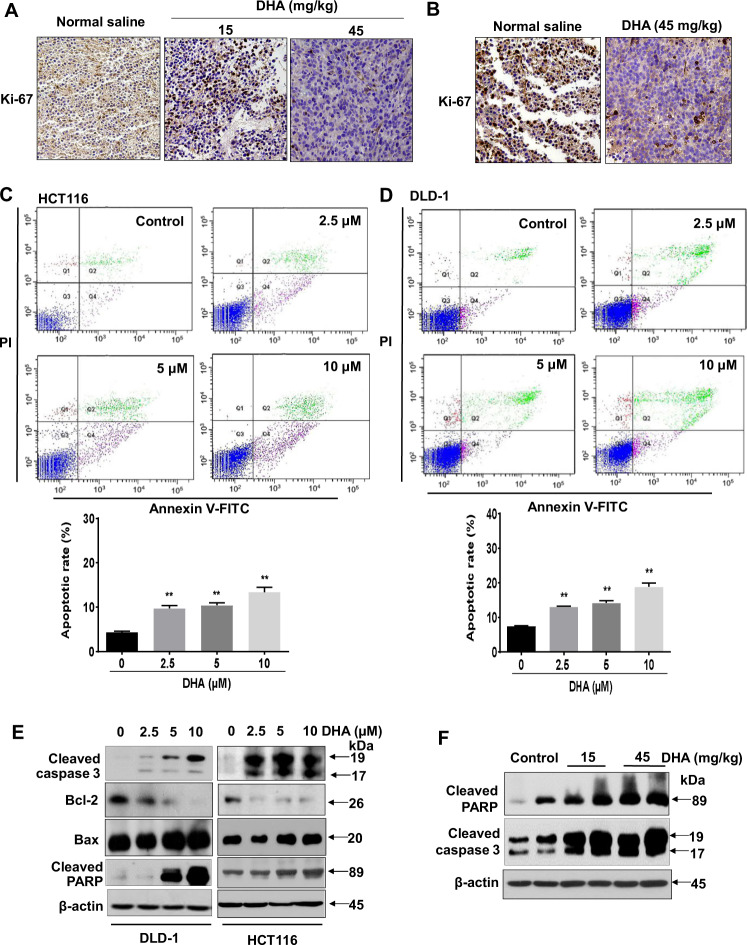
Dihydroartemisinin reduces Ki-67 expression and induces apoptosis in CRC, both in vitro and in vivo. Immunohistochemistry staining showing the expressions of Ki67 in the tumors of the (**A**) xenograft mouse models and (**B**) orthotopic mouse models after dihydroartemisinin (DHA) treatments at the indicated doses. **C, D** Percentages of apoptotic cells after dihydroartemisinin (DHA) treatments at the indicated concentrations. The data are shown as means ± SEM. *n* = 3 independent experiments, *p < 0.05, **p < 0.01 compared with control**. E** Western blot showing the expressions of cleaved caspase-3, Bcl-2, Bax, and cleaved PARP in the CRC cells after dihydroartemisinin (DHA) treatments at the indicated concentrations. **F** Western blot showing the expressions of cleaved caspase-3 and cleaved PARP in the xenograft tissues of the DLD-1-bearing xenograft mouse models after dihydroartemisinin (DHA) treatment at the indicated doses.

Reduced energy availability will reduce cancer growth. Indeed, dihydroartemisinin also reduced the cancer-cell proliferation as indicated by the reduced expressions of proliferative marker Ki67 in the tumors of the xenograft mouse model (Fig. 6A) and orthotopic mouse model (Fig. 6B). Our data suggest that dihydroartemisinin reduces energy production in CRC and inhibits CRC cell proliferation. It has been reported that reduced mitochondrial ATP production is a crucial early step in apoptosis [18]. Modulating ATP availability has been considered as an important strategy in inducing cell death [19]. As expected, dihydroartemisinin treatments significantly increased the percentages of apoptotic cells (Fig. 6C, D), and the expressions of apoptotic markers both in vitro (Fig. 6E) and in vivo (Fig. 6F).

## Discussion

Our study clearly shows that c-Myc expression is higher in CRC than in normal colon epithelial cells, the expression of c-Myc in CRC is also directly correlated with the staging of the cancer. Dihydroartemisinin treatment significantly reduces the expressions of c-Myc and its downstream targets ACC, FASN, CPT-1, and MCAD, which changes the lipid metabolism and the metabolic phenotypes in CRC, reduces energy production, cell proliferation, and induces cancer-cell death.

Clinical studies show that CRC with c-Myc overexpression has a significant higher frequency of metastasis and is associated with poor prognosis [3–7]. However, other studies show that expression of c-Myc in primary cancer is significantly correlated with improved survival and better prognosis [20, 21]. The inconsistent reports on the pathogenic roles of c-Myc in CRC deserve further clinical investigation. Other gene mutations in CRC or the association with other complications during diagnosis may be the confounding factors that affect the results. Nevertheless, mounting of experimental evidence shows that c-Myc is an oncogene that regulates cellular metabolism and hence cellular growth [11, 12, 22].

Many studies have documented the role of c-Myc in driving a transcriptional program that promotes cell metabolism [23]. Examples of the c-Myc direct target genes involved in metabolism are lactate dehydrogenase A (LDHA) involved in glycolysis [24] and ornithine decarboxylase (ODC) involved in polyamine synthesis [25]. However, whether ACC, FASN, CPT1, and MCAD are the direct targets of c-Myc are less studied [26]. Many canonical c-Myc target genes involved in metabolism have conserved high-affinity MYC-consensus E-box-binding sites in their proximal promoters [27], or nonconsensus sites to which MYC binds with lower affinities when MYC levels are high and deregulated [28]. In the future, we will examine whether these consensus E-box-binding sites and nonconsensus sites are present in the promoters of ACC, FASN, CPT1, and MCAD genes and examine their functional roles, with chromatin-immunoprecipitation analyses, next-generation sequencing, and other functional studies. Recently, a multiomics-based study with metabolomics, target sequencing of cancer-related genes, transcriptomic, and methylated DNA-immunoprecipitation sequencing has been done on the clinical CRC samples. The analysis reveals that c-Myc expression induces at least 215 metabolic reactions by changing the expression levels of metabolic genes and transporter genes [12]. The c-Myc-driven cancers are characterized by increased nutrient uptake, glycolysis, glutaminolysis, and elevated fatty acid and nucleotide synthesis [29, 30]. Among all these metabolic pathways, the impact of c-Myc activity on lipid metabolism is the most prominent. Studies report that in c-myc-null cells, mitochondria become dysfunctional, fatty-acid beta-oxidation is repressed, and ATP production in the cells is attenuated [31, 32]; however, the role of c-Myc in CRC metabolism is less studied. Our study evidently shows that dihydroartemisinin modulates the metabolism in CRC. Since CRC needs more energy than normal cells to support their growth, inhibition of the c-Myc-mediated energy production will have a greater detrimental consequence in CRC than in normal tissues, which favors the development of dihydroartemisinin as an anti-CRC therapeutic agent.

Although c-Myc is a well-known CRC driver, inhibiting c-Myc function is a big challenge. One of the challenges in directly inhibiting c-Myc activity is the primary active site in c-Myc remains unknown [33]. The c-Myc protein displays a predominantly intrinsically disordered structure and lacks a binding pocket for the design of small molecule to inhibit its transcriptional activity [34], which limits the development of small-molecule antagonist for c-Myc. Interfering the binding of Myc/Max dimerization to inhibit the Myc activity is a strategic approach. 10058-F4 and its active analogs bind specifically to monomeric Myc and interact with the H2/leukine zipper domain [35]. However, preclinical study shows that 10058-F4 has no significant effect on tumorigenesis [36]. Other attempts using Myc-antisense technology and Myc-dominant negative mutant for cancer treatment have been made; however, Phase-I and -II clinical trials were discontinued [37]. On top of these difficulties, c-Myc is a critical transcription factor in many biological functions. It mediates the transcriptional responses to various cellular signaling that drives cell growth and proliferation and impacts differentiation, survival, and pluripotency [38]. Therefore, preferentially inhibiting c-Myc in CRC cells, but not other cell types, becomes a critical issue for the design of c-Myc-targeted therapeutics. Inhibitors that target c-Myc activity in cancer cells may also inhibit c-Myc activity in other cell types that leads to adverse side effects. Currently, no clinical frontline drug is targeting c-Myc protein for CRC treatment. Given that the late-stage CRC has an elevated c-Myc expression, the c-Myc targeting property of dihydroartemisinin may be advantageous for treating the late-stage CRC. Indeed, our study also shows that dihydroartemisinin has a higher inhibitory potency in late-stage CRC when compared with the early-stage CRC. Nevertheless, in-depth acute and chronic toxicity tests have to be done to further suggest its safety for late-stage CRC treatment.

Dihydroartemisinin belongs to a family of compounds derived from the natural sesquiterpene lactone artemisinin (Coartem^®^/Riamet^®^). It is a cyclic endoperoxide. It generates reactive oxygen species (ROS)-like superoxide anions, hydroxyl radicals, and carbon-centered radicals when its endoperoxide bridge is activated by ferrous iron [39]. The generated ROS and carbon-centered radicals induce oxidative damage to membrane lipids and DNA in cancer cells, which will lead to the ROS-dependent apoptosis with the activation of pro-apoptotic Bcl-2 family member Bax and caspase-activation [40]. A study has demonstrated that dihydroartemisinin induces apoptosis in CRC through the mitochondria-dependent pathway in which dihydroartemisinin decreases mitochondrial-membrane potential, increases ROS accumulation, and release of cytochrome c from the mitochondria [41]. The relationship between ROS production and lipid metabolism in dihydroartemisinin-treated CRC has not been studied. Our study shows that dihydroartemisinin changes the lipid metabolism in CRC; however, whether the dysregulated lipid metabolism would further enhance the ROS production caused by the activation of endoperoxide bridge is yet to be known.

In conclusion, our study clearly shows that c-Myc expression in CRC increases as the cancer stage advances. Dihydroartemisinin reduces c-Myc expression and inhibits its transcriptional activities in CRC, hence changes the lipid metabolism in the cells, reduces energy production, and induces cell death. More importantly, we are the first to demonstrate that dihydroartemisinin exhibits a higher inhibitory potency in late-stage than in the early-stage CRC. Our study provides a rationale for the development of dihydroartemisinin as therapeutic agent for the treatment of late-stage CRC by targeting c-Myc.

## Materials and methods

### Reagents and chemicals

Dihydroartemisinin (KPC Pharmaceuticals Inc.) was dissolved in DMSO. Chemicals, including 3-[4,5-dimethylthiazol-2-yl]-2,5-diphenyltetrazolium bromide (MTT), fluorouracil (5-Fu), MG132, palmitic acid, oleic acid, and stearic acid, were purchased from Sigma-Aldrich. D-Luciferin (115144-35-9) was purchased from Cayman Chemical. Primary antibodies against c-Myc (#9402), FASN (#3180), ACC (#3676), cleaved caspase 3 (#9661), cleaved PARP (#5625), Bcl-2 (#15071), Bax (#2772), and β-actin (#12262) were purchased from Cell Signaling Technology. Primary antibodies against Ki67 (sc-23900), MCAD (sc-365109), and SCD1 (sc-14719) were purchased from Santa Cruz Biotechnology. Primary antibody against CPT-1A (DF12004) was obtained from Affinity Biosciences. All the secondary antibodies were from Santa Cruz Biotechnology. Human CRC tissue array (CO702d) was purchased from US BIOMAX.

### Cell culture

The CRC cell lines SW480, SW620, DLD-1, COLO205, HCT116, SW1116, and normal colon-cell lines IEC-6 and CCD841CoN were purchased from the American Type Culture Collection (ATCC) and were authenticated. The cell lines were tested for mycoplasma contamination. HCT116 cells with stable overexpression of firefly luciferase (Fluc) and enhanced green fluorescent protein (eGFP) were purchased from Imanis Life Sciences. All the cell lines were cultured following the company’s protocols.

### Cell-viability assay

Cell viability was examined by MTT assay. The absorbance of each well was measured at wavelength 570 nm in a microplate reader. Cell viability was calculated according to the absorbance of each well with the following formula: cell viability (%) = [(A570 sample - A570 blank)/(A570 control - A570 blank)] × 100%, where A570 sample, A570 blank, and A570 control stand for the absorbance of dihydroartemisinin-treatment group, blank group (no cells), and control group (vehicle), respectively.

### Colony-formation assay

Cells were seeded in a 6-well plate at the density of 500 cells per well and cultured for 24 h at 37 °C, before dihydroartemisinin (2.5 or 5 µM) treatments. Vehicle served as control. The medium containing dihydroartemisinin was replenished every three days. After 15 days, the cells were washed with PBS, fixed for 15 min with 4% paraformaldehyde at room temperature, and then stained with 0.1% crystal violet for 30 mins. Cells were washed three times after the crystal violet solution being discarded. The plates were air-dried, and the visible colonies were photographed.

### CRC-bearing xenograft mouse model

Balb/c nude mice aged 5–6 weeks were purchased from the Laboratory Animal Services Centre of The Chinese University of Hong Kong. Mice were housed in aseptic laminar-flow cabinets with a 12-h light–dark cycle and free access to standard rodent chow and sterile water. CRC cells (2 × 10^6^ DLD-1, HCT116, HCT116^c-myc+^, or HCT116^EV^) in 100 μL of PBS were subcutaneously inoculated into the right flank of nude mice. When the tumor volumes reached 100 mm^3^, the mice were randomly assigned into groups and were treated with dihydroartemisinin (15 mg/kg or 45 mg/kg) once every day for 21 days by intraperitoneal route. The tumor volume was measured using a caliper every three days and calculated with the following formula: V = L × W^2^/2, where L and W stand for the length and width of tumor, respectively. No blinding was done. After the treatment, mice were sacrificed, and tumors were dissected and weighted.

### CRC-bearing orthotopic mouse model

Athymic Balb/c mice aged 5–6 weeks were purchased from the Experimental Animal Centre of The Chinese University of Hong Kong. Mice were maintained in laminar-flow cabinets with free access to standard rodent chow and sterile water. The orthotopic CRC tumor model was constructed as described. Briefly, mice were anesthetized and received laparotomy with the cecum exposing and exteriorizing. HCT116-Fluc-Neo/GFP-puro cells (5 × 10^5^) were suspended in 40 ul of PBS/Matrigel (1:1) and inoculated into the submucosa of mice cecum with a 29-gauge needle. One week after cell inoculation, the mice were randomized into two groups. Mice in the control group were treated with vehicle and mice in treatment group were injected with dihydroartemisinin (45 mg/kg) by intraperitoneal route, respectively. Bioluminescence signal was monitored every six days using the IVIS 2000 Imaging System coupled with Living Imaging Software (Caliper). D-luciferin substrate (150 mg/kg) was intraperitoneally injected into the mice 10 mins before the bioluminescence-signal measurement. No blinding was done. Mice were sacrificed 24 days after the treatment and the tumors were dissected and fixed in 4% paraformaldehyde.

### RNA extraction and real-time PCR (RT-PCR) reaction

Total RNA of CRC cells was extracted using Trizol reagent (Invitrogen) according to the manufacturer’s protocol. cDNA was synthesized in a total volume of 20 ul using a cDNA reverse transcription reaction kit (TAKARA). RT-PCR was performed with a PrimeScript™ RT reagent Kit (TAKARA) using a Viia7 RT-PCR machine (Thermo Fisher Scientific). Primers for c-Myc were 5′-AAAGGCCCCCAAGGTAGTTA-3′ (forward), 5′-GCACAAGAGTTCCGTAGCTG-3′(reverse); for FASN: 5′-CTTCCGAGATTCCATCCTACGC-3′(forward), 5′-TGGCAGTCAGGCTCACAAACG-3′(reverse); for ACC1: 5′-GCTCCTTGTCACCTGCTTCT-3′(forward), 5′-AAGGCCAAGCCATCCTGTA- 3′(reverse); for CPT1: 5′-ATCAATCGGACTCTGGAAACGG-3′(forward), 5′-TCAGGGAGTAGCGCATGGT-3′(reverse); for MCAD: 5′-GGAAGCAGATACCCCAGGAAT-3′(forward), 5′-AGCTCCGTCACCAATTAAAACAT-3′(reverse); for GAPDH: 5′-TTGGTATCGTGGAAGGACTCA-3′(forward), 3′-TGTCATCATATTTGGCAGGTT- 5′(reverse). All the mRNA expressions were normalized to that of GAPDH.

### Western blot assay

After treatments, CRC cells or tumors were collected and lysed for 30 mins on ice with lysis buffer. Proteins were separated in a 6–12% sodium dodecyl sulfate polyacrylamide gel (SDS-PAGE) and transferred onto a polyvinylidene difluoride (PVDF) membrane preactivated by methanol. The membrane was blocked for 2 h at room temperature with 5% nonfat milk powder dissolved in TBST (0.1% Tween-20 in TBS) and then incubated with the respective primary antibodies for 12 h at 4 C. The secondary antibodies were diluted (1/10000) in TBST containing 5% milk and incubated for 1 hr at room temperature. The immune-reactive targets were detected by an ECL Western Blotting Substrate Kit (Thermo Fisher Scientific). The band density was analyzed by Image J software and normalized with internal control.

### siRNA transfection

CRC cells were transfected with siRNA against c-Myc (Santa Cruz Biotechnology) with Lipofectamine 3000 (Lipofectamine™ RNAiMAX) transfection reagents according to the manufacturer’s instruction (Invitrogen). Twenty-four hours after siRNA transfection, cells were treated with or without dihydroartemisinin for another 24 h. Vehicle served as control.

### c-Myc overexpression

CRC cells were transfected with 2 μg of pcDNA3-cmyc plasmid (Addgene) encoding human c-Myc using Lipofectamine 3000 transfection reagents. G418 antibiotic (Thermo Fisher Scientific) was used to establish the stable CRC cells that overexpress c-Myc. RT-PCR and Western blot assays were used to validate the c-Myc overexpression in these cells.

### Dual-luciferase reporter assay

HCT 116 and DLD-1 cells were cotransfected with c-Myc reporter plasmid and pRL-TK-Renilla-luciferase plasmid using Lipofectamine 2000 (Thermo Fisher Scientific, USA). After 24 h, cells were treated with vehicle or dihydroartemisinin at the indicated concentration for 48 h, and then lysed with 200 μL of lysis buffer (Beyotime Biotechnology, China). A 20-μL aliquot of the cell lysate was subjected to a luciferase assay using Dual Luciferase Assay Kit (Beyotime Biotechnology). c-Myc reporter activity was detected using EnVision Mutilabel Reader. Relative luciferase activity was normalized to that of Renilla luciferase.

### UPLC/MS-based lipidomics

Dihydroartemisinin at the respective IC_30_ concentrations was used to treat the different CRC cell lines for 48 h. Vehicle served as control. The amount of protein in each sample was quantified by Pierce BCA Protein Assay (Thermo Fisher Scientific). The lipid in the samples was extracted by Folch reagent and dried under gentle nitrogen stream and kept at −80 C. The dried lipid samples were dissolved in 200 µl of mixed solution, including isopropanol (10%) and 10 mM of NH4·formate (pH4, 90%) with sonication. The samples were then centrifuged, and the supernatant was collected for UPLC/MS analysis. A commercial chemical deuterium-labeled hexadecanoic-15,15,16,16,16-d5 acid was used as an internal standard. The precision of UPLC–MS method was performed. Total lipids were analyzed using the Agilent 6540 UHD Accurate-Mass Q-TOF LC/MS mass spectrometer connecting with an Agilent 1290 Infinity UHPLC via an electrospray-ionization source. Global lipidomics analysis was performed by Mass Profiler Professional (MPP) software (Version 2.2, Agilent Technologies) connecting a lipid metabolites database, the data were processed to extract the ion through the Mass Hunter Workstation by comparing the retention time, intensity in the apex of chromatographic peak, and mass. For targeted metabolite analysis, commercial standards of palmitic acid, oleic acid, and stearic acid (Sigma-Aldrich) were used for quantification.

### Acetyl-CoA detection

The acetyl-CoA levels in CRC cells were examined with acetyl-CoA assay kit (Abcam) following the manufacturer’s protocol. Acetyl-CoA concentrations were calculated according to a standard curve and protein concentrations were determined by Pierce BCA Protein Assay Kit (Thermo Fisher Scientific).

### ATP measurement

The ATP levels were detected using an enhanced ATP Detection Kit (Beyotime Biotechnology, China) following the manufacturer’s protocol. CRC cells or tumor samples were lysed using ATP lysis and centrifuged at 12,000 *g* for 5 mins. The supernatant was collected for ATP measurement using an illuminometer reader. Protein concentration of each sample was determined by Pierce BCA Protein Assay Kit (Thermo Fisher Scientific).

### Measurement of the oxygen-consumption rate (OCR) and extracellular acidification rate (ECAR) in CRC cells

The OCR measurement of CRC cells was measured by Seahorse XF24 Extracellular Flux Analyzer (Agilent) and was performed according to the manufacturer’s protocol. Briefly, CRC cells were seeded at 9000 cells per well in XF24 cell culture plate in 100 μl of culture medium and incubated for 20 hr at 37 °C and 5% CO_2_, and then treated with 10 μM dihydroartemisinin for 24 h prior to the OCR assay. Then, the cell culture medium was replaced by pH 7.4 XF assay medium supplemented with 1 mM pyruvate, 2 mM glutamine, and 10 mM glucose, and incubated at the incubator without supplied CO_2_ for 1 h before the completion of probe-cartridge calibration. Basal respiration was measured in the XF assay medium without oligomycin, and mitochondrial function was measured by injecting oligomycin (1.5 μM), FCCP (1 μM), and rotenone (0.5 μM) mix with antimycin A (0.5 μM) as indicated.

For ECAR measurement, CRC cells were treated with 10 μM dihydroartemisinin for 24 h prior to ECAR assay. On the day of ECAR measurement, cell culture medium was replaced by pH 7.4 XF assay medium supplemented with 2 mM glutamine and incubated at the incubator without supplied CO_2_ for 1 h before the completion of probe-cartridge calibration. Basal ECAR was measured in the XF assay medium without glucose, and glycolysis was measured by injecting glucose (10 mM), oligomycin (1 μM), and 2-deoxyglucose (50 mM) from XF24 reagent ports as indicated. After the test, the total protein in each well was measured by BCA method and the data were normalized on proteins.

### Flow cytometry

CRC cells were treated by dihydroartemisinin or vehicle for 48 h. Cells were collected and stained with annexin V-FITC & PI staining solution following the manufacturer’s protocol (Annexin V-FITC/PI Apoptosis Detection kit, BD Bioscience). After 15 min of incubation, cells were immediately analyzed for apoptosis by FACScan flow cytometry (Becton Dickinson).

### Immunohistochemistry (IHC) analysis

The tumor tissues were dissected and fixed for 24 h in 4% paraformaldehyde before sectioning. For the staining, the slides were incubated with HRP-conjugated secondary antibodies for 1 h at room temperature and subsequently exposed to diaminobenzidine peroxidase substrate for 5 min. The slides were counterstained with hematoxylin and eosin. Image analysis was performed by Image-Pro Plus (version 6.0). Staining procedures of the human CRC tissue array slides followed the company’s instruction.

### Measurement of mitochondrial-membrane potential

CRC cells were seeded in a 6-well plate (3 × 10^5^/well) and grown for 24 h. After transfection with siRNA-targeting c-Myc for 6 h, cells were treated with or without dihydroartemisinin at 10 μM for 48 h. Cells then were collected and washed twice with cold PBS. Subsequently, cells were stained with a commercial JC-1 staining kit (Beyotime Biotechnology) and analyzed with flow cytometry.

### Statistical analysis

We used SPSS software to perform statistical analysis of the data, one-way analysis of variance (ANOVA) to examine the variance within each group and the significant difference between groups with *p < 0.05, **p < 0.01, and **p < 0.001. The data are shown as mean ± SE, *n* = 3 independent experiments.

## Supplementary information

Supplementary Figures

Original WB film corrected (not labeled in red)

checklist

ccids-author-contribution form

Agreement on the change of authorship

## Data Availability

It does not apply for the paper.
