# Supplementary material for: Dihydroartemisinin is potential therapeutics for treating late-stage CRC by targeting the elevated c-Myc level
Source: Cell Death Dis. 2021 Nov 5;12(11):1053. doi: 10.1038/s41419-021-04247-w (PMC8571272; doi:10.1038/s41419-021-04247-w)
Supplement: Supplementary file 1 — Supplementary Figures [file 41419_2021_4247_MOESM1_ESM.pdf]

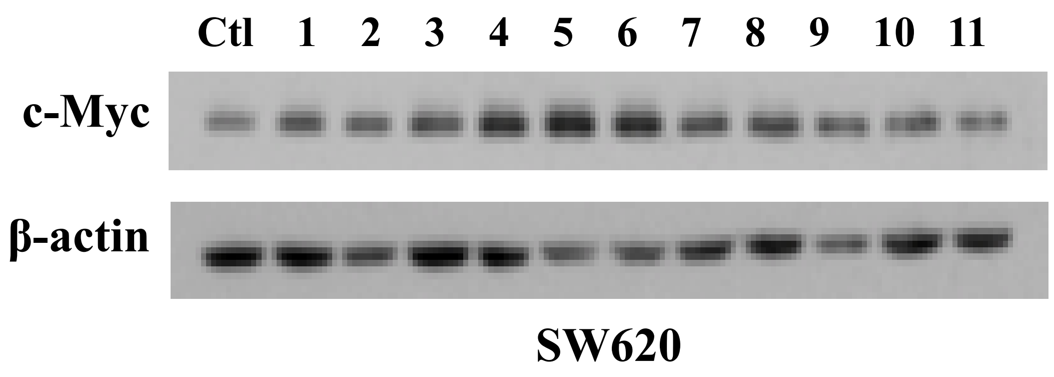

Supplementary Figure S1 Screening of herbal compounds for their effects on c-Myc expression in CRC cells. Western blot showing the protein expressions of c-Myc in SW620 cells after the compound treatments at their respective IC^{40} concentrations. 1, Baicalein (15µM); 2, Dihydrotanshinone (1.8µM); 3, Caffeic acid (6.6µM); 4, Capsaicin (37µM); 5, Apigenin (15.6µM); 6, Quercetin (12.0µM); 7, Hesperidin (64µM); 8, Fucoxanthin (5µM); 9, Schisandrin B (50µM); 10, Catechin (3.5µM); 11 Protocatechin acid (5µM), vehicle served as control (Ctl).

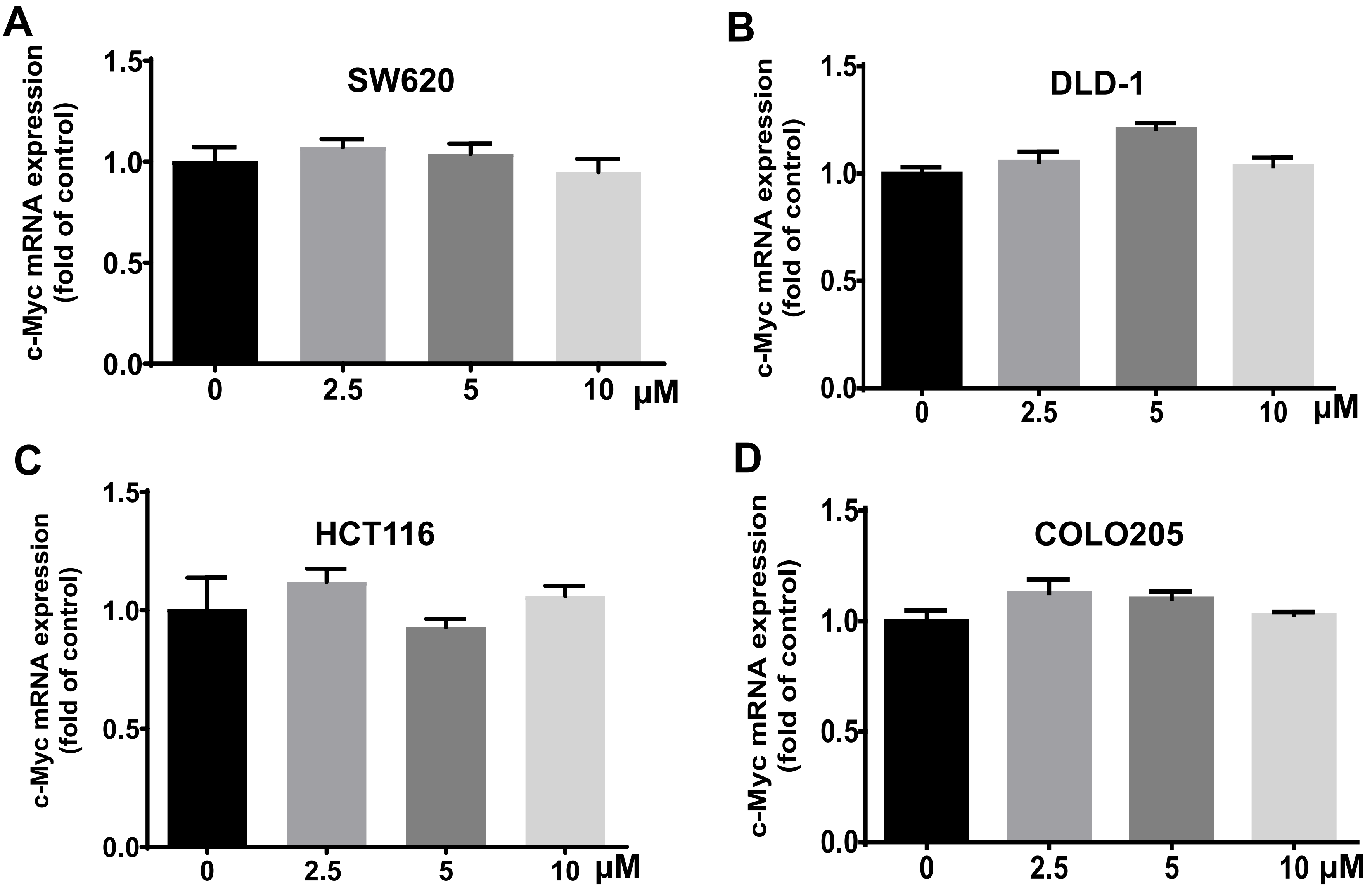

Supplementary Figure S2 c-Myc mRNA levels in **(A)** SW620, **(B)** DLD-1, **(C)** HCT116 and **(D)** COLO205 cells after dihydroartemisinin (DHA) treatments for 48hr at the indicated concentrations. The data are shown as means ± SEM. n = 3 independent experiments.

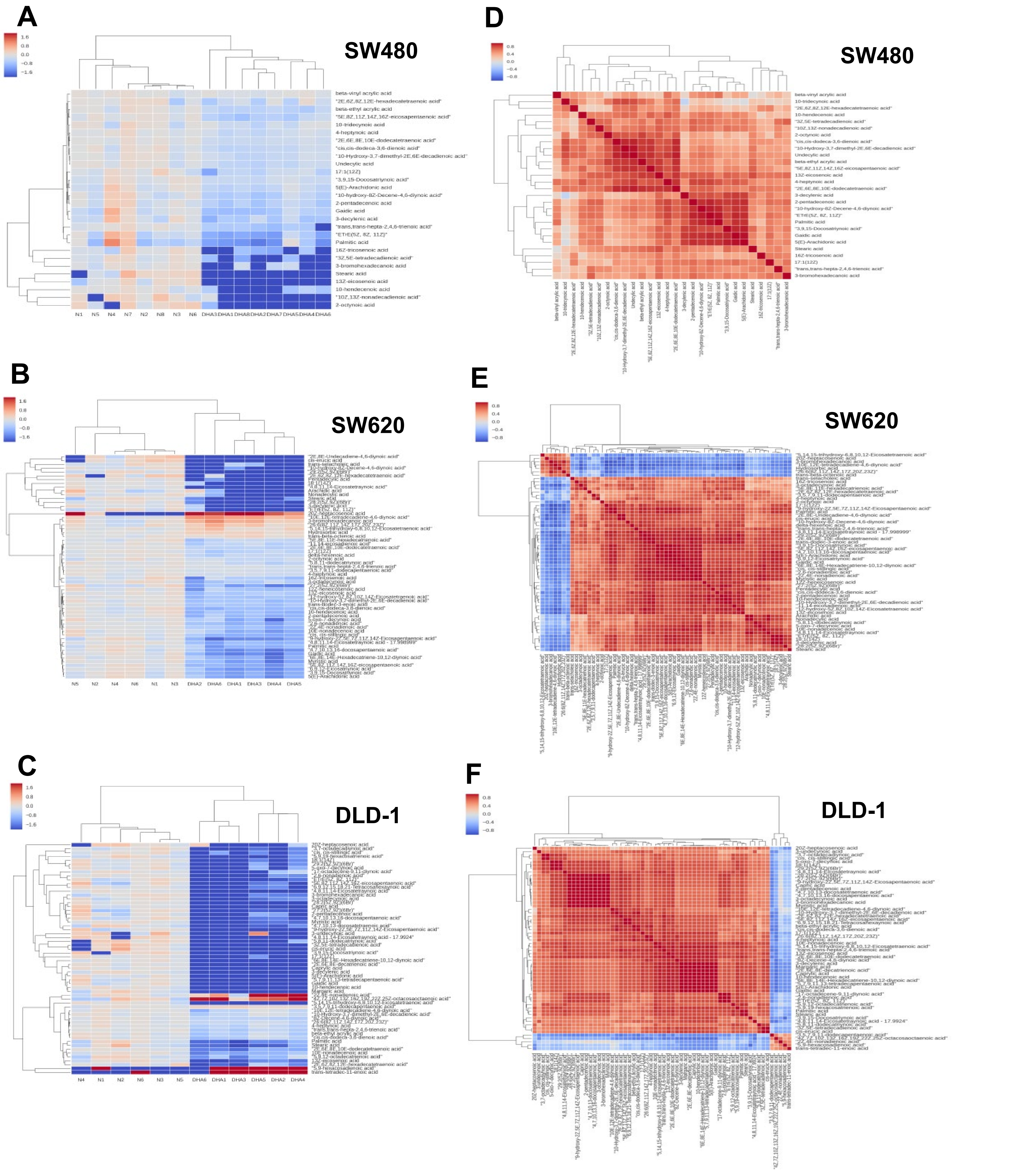

Supplementary Figure S3 (A-C) Heat maps and hierarchical clustering for the metabolites that are significantly different between control and dihydroartemisinin-treated samples. The colour is proportional to the fold change of each metabolite (red showing up-regulated; blue showing down-regulated). Rows: lipid metabolites; Columns: CRC samples. **(D-F)** The correlation analysis of differential metabolites in control and dihydroartemisinin-treated samples

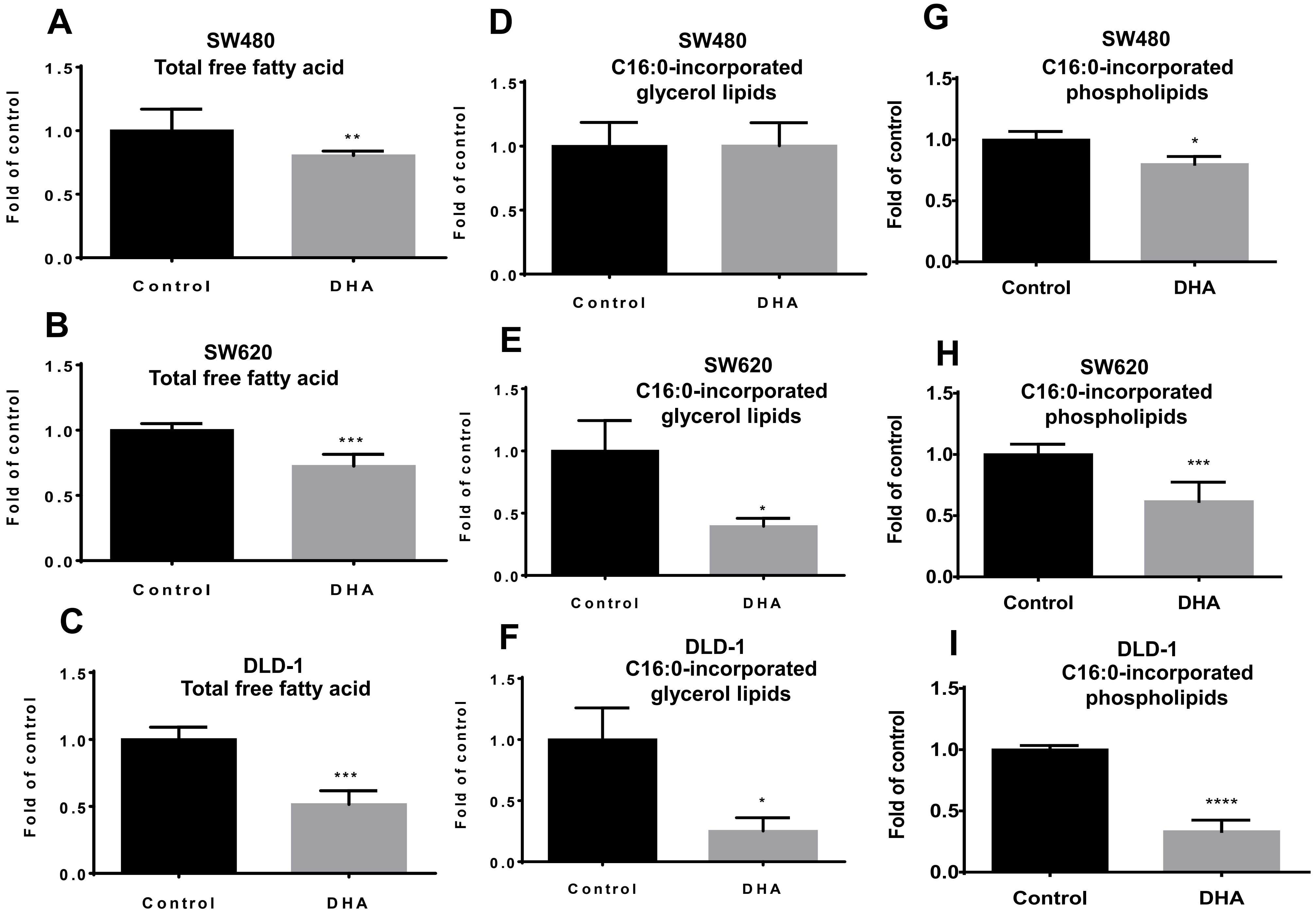

Supplementary Figure S4 (A-C) Total free fatty acid levels, **(D-F)** C16:0-incorporated glycerol lipid levels, and **(G-I)** C16:0-incorporated phospholipids in CRC cells after dihydroartemisinin (DHA) treatments. The data are shown as means ± SEM. n = 3 independent experiments, *p<0.05, *p< 0.01, ***p< 0.001 compared to control.

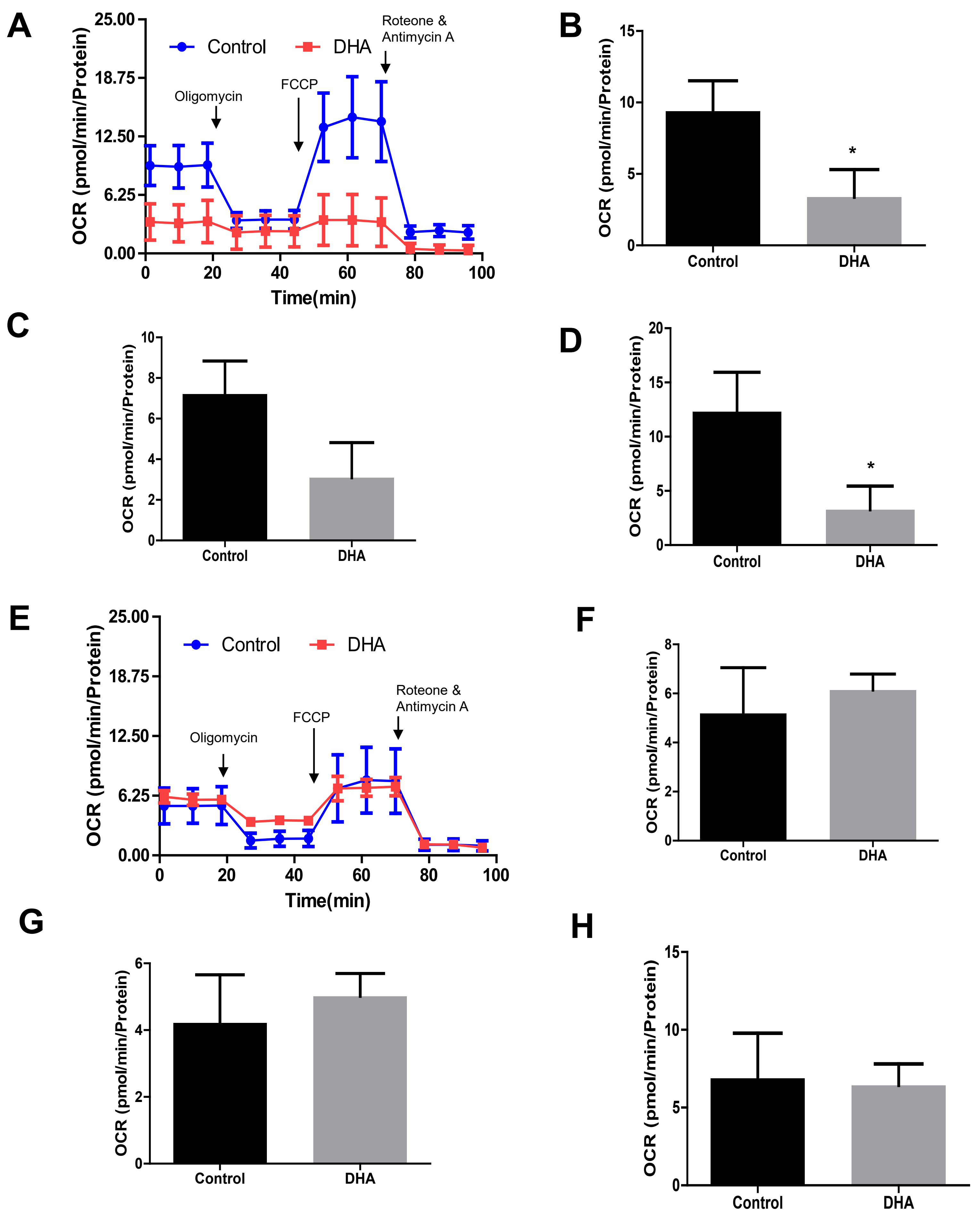

Supplementary Figure S5 (A) The oxygen consumption rate (OCR), **(B)** quantification of OCR, **(C)** basal respiration and **(D)** maximal respiration of the DLD-1 cells upon dihydroartemisinin (DHA, 10μM) challenge, vehicle served as control. **(E)** The oxygen consumption rate (OCR) and **(F)** quantification of OCR, **(G)** basal respiration and **(H)** maximal respiration of the c-Myc-knockdown DLD-1 ($DLD1^{c\text{-}Myc\text{-}siRNA}$) cells upon dihydroartemisinin (DHA, 10μM) challenge, vehicle served as control. The data are shown as means ±SEM. n = 3 independent experiments, *$p<0.05$ compared to control.

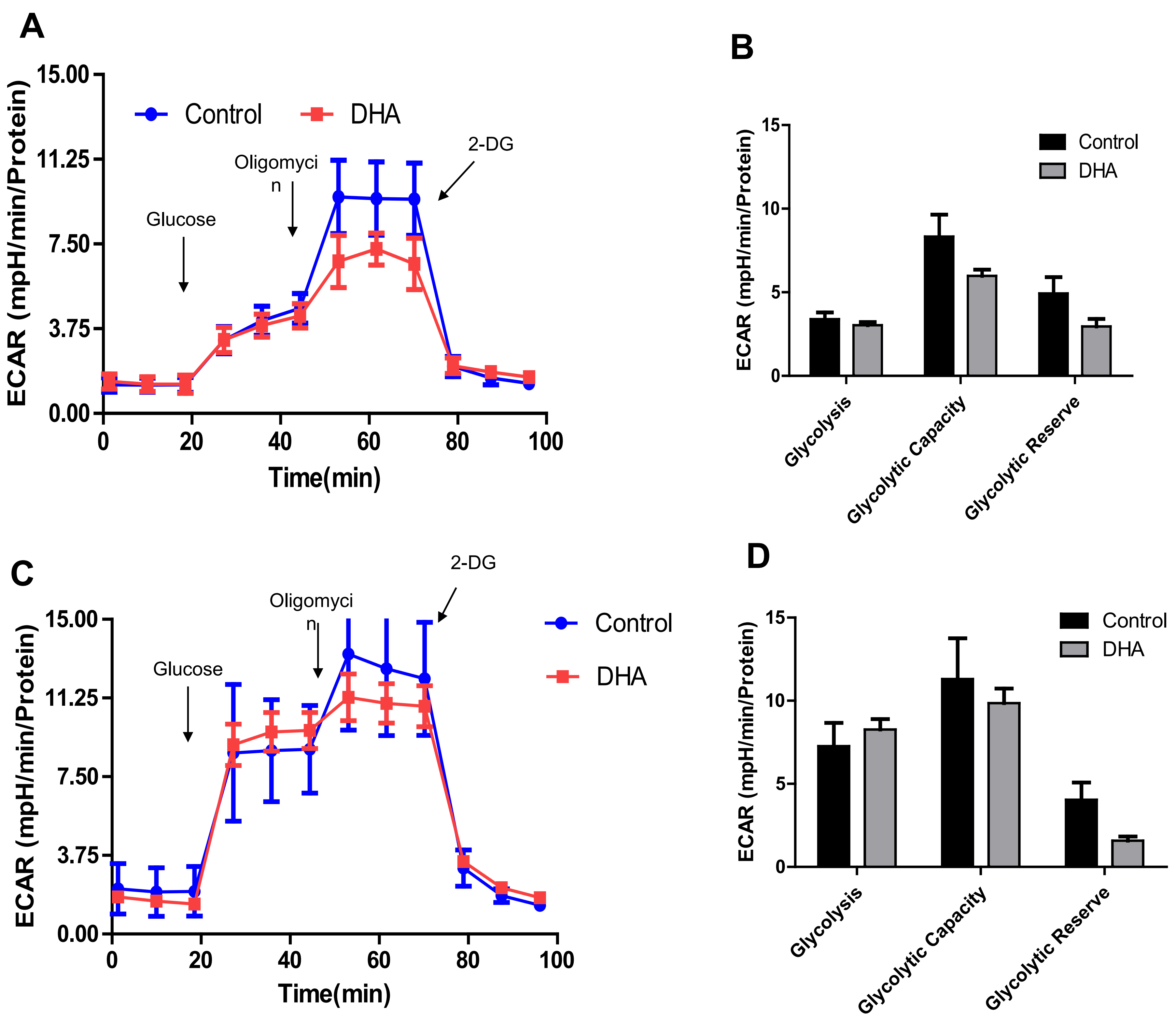

Supplementary Figure S6 (A) The extracellular acidification rate (ECAR), **(B)** quantification of ECAR, glycolysis, glycolytic capacity, glycolytic reserve in HCT116 cells upon dihydroartemisinin (DHA, 10μM) challenge. **(C)** The ECAR, **(D)** quantification of ECAR, glycolysis, glycolytic capacity, glycolytic reserve in DLD-1 cells upon dihydroartemisinin (DHA, 10μM) challenge. The data are shown as means ±SEM. n = 3 independent experiments.
