# Supplementary material for: Dihydroartemisinin is potential therapeutics for treating late-stage CRC by targeting the elevated c-Myc level
Source: Cell Death Dis. 2021 Nov 5;12(11):1053. doi: 10.1038/s41419-021-04247-w (PMC8571272; doi:10.1038/s41419-021-04247-w)
Supplement: Supplementary file 2 — Original WB film corrected (not labeled in red) [file 41419_2021_4247_MOESM2_ESM.pdf]

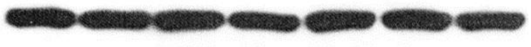

Figure 1C beta-actin

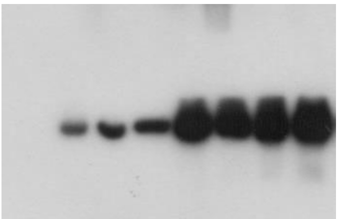

Figure 1C c-Myc

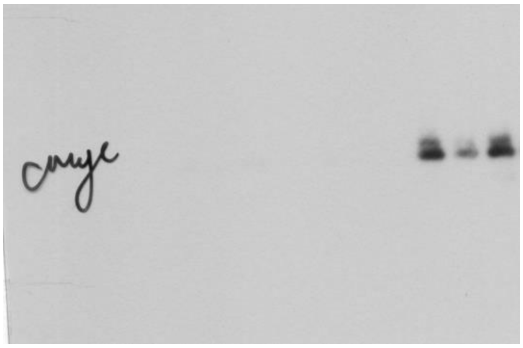

Figure 1D cMyc DLD1

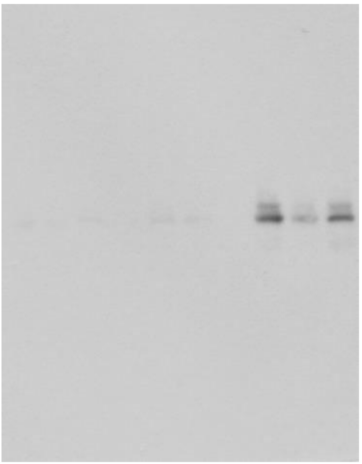

Figure 1E cMyc HCT116

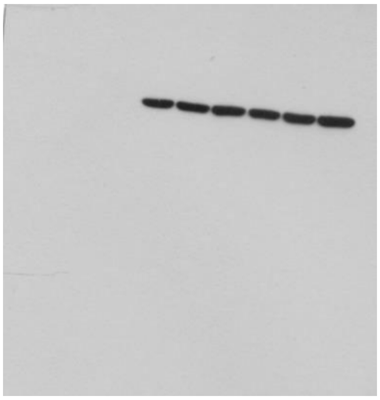

Figure 1D and 1E beta-actin

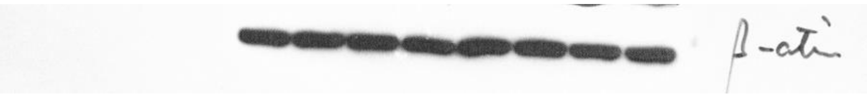

Figure 2A beta-actin SW480 and DLD-1

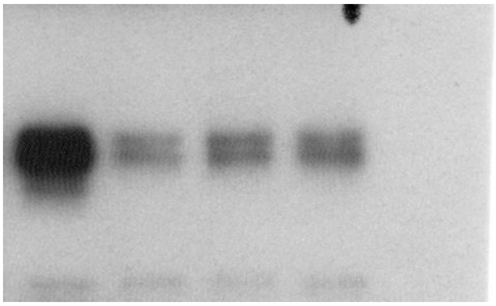

Figure 2A cMyc DLD-1

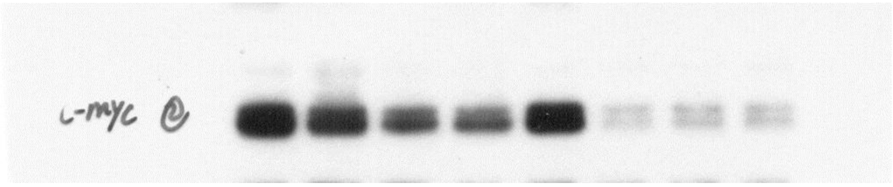

Figure 2A cMyc SW480 and HCT116

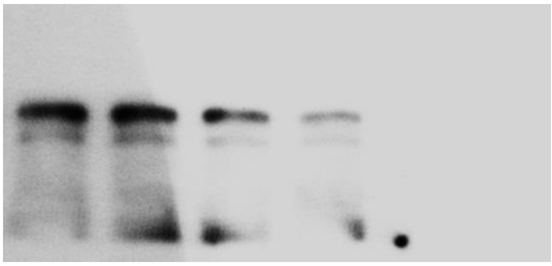

Figure 2A cMyc SW620

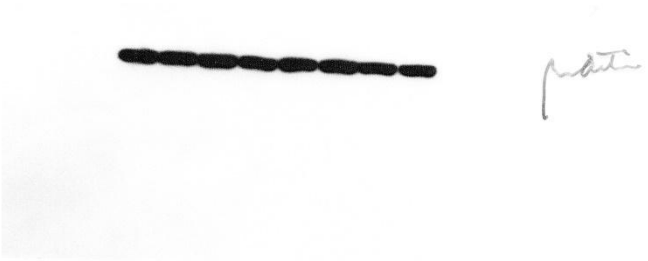

Figure 2A beta-actin HCT116 and SW620

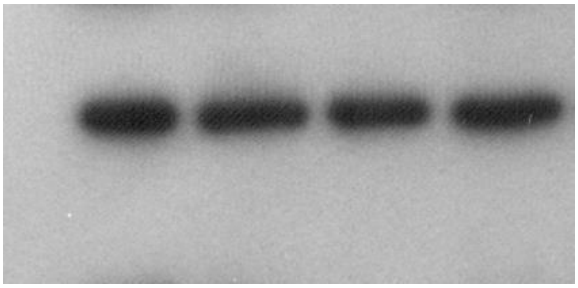
Figure 2B beta-actin

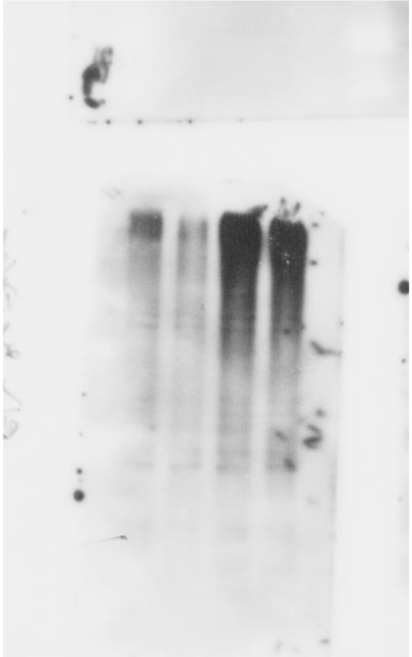
Figure 2B Ubiquitinated protein

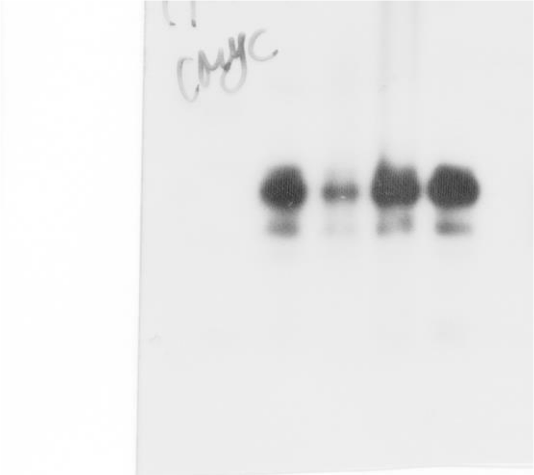

Figure 2C cMyc HCT116

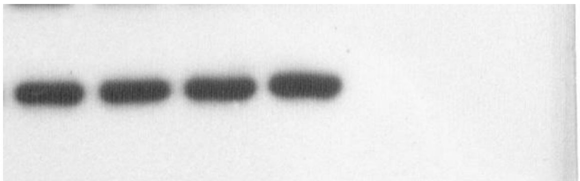
Figure 2C beta-actin

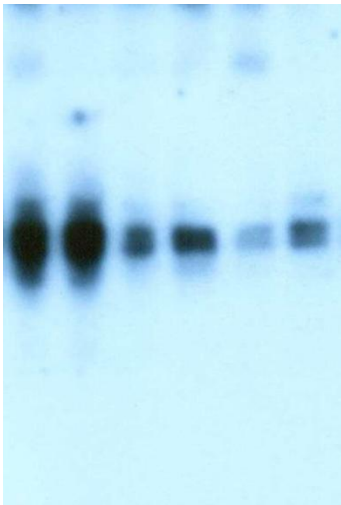

Figure 3G cMyc tumor

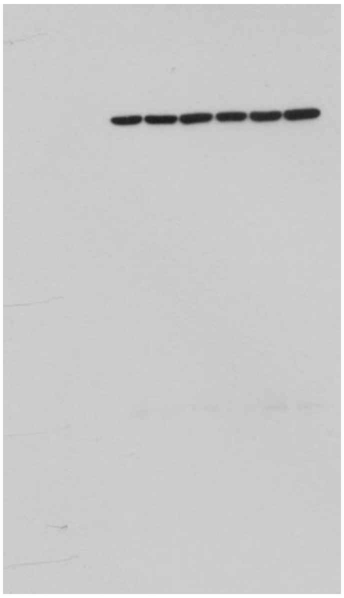

Figure 3G beta-actin

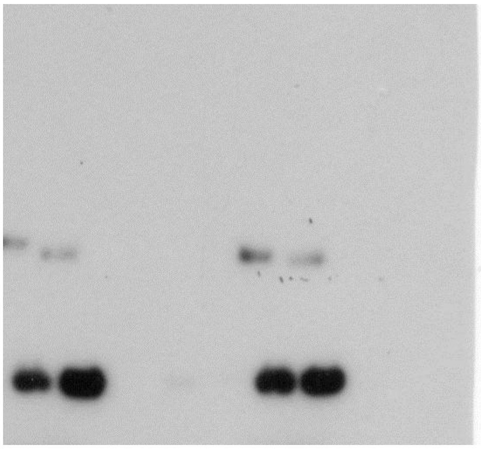

Figure 4B ACC DLD1 and HCT116

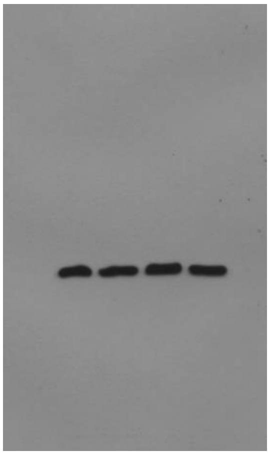

Figure 4B beta-actin DLD1 and HCT116

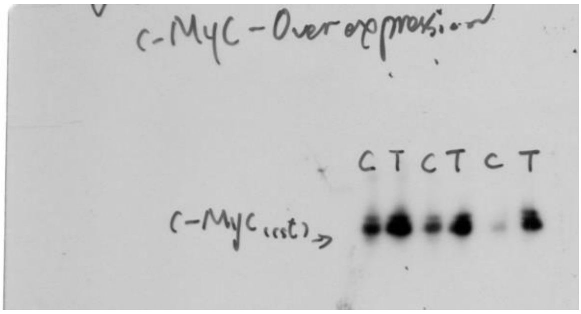

Figure 4B cMyc overexpression HCT116 and DLD-1

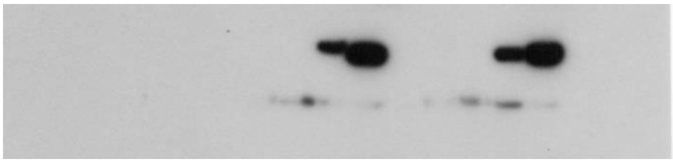

Figure 4B CPT1 DLD-1 and HCT116

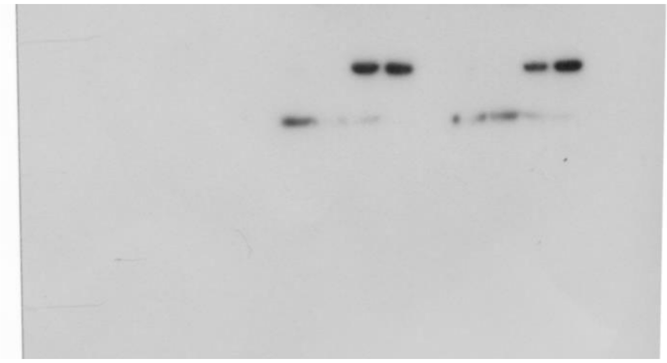

Figure 4B FASN DLD-1 and HCT116

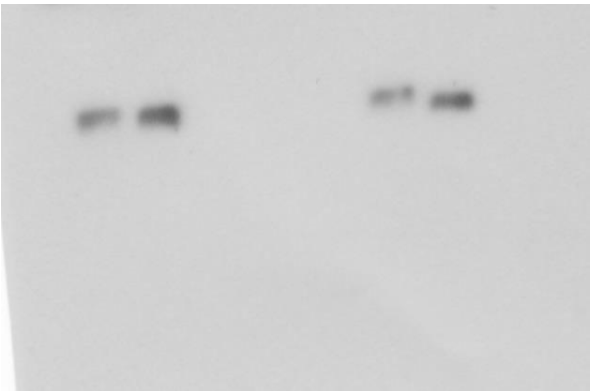

Figure 4B MCAD DLD-1 and HCT116

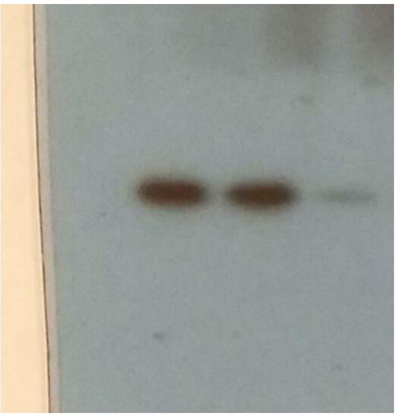
Figure 4C ACC HCT116

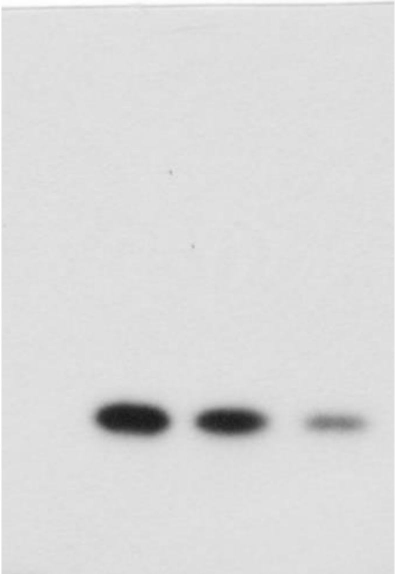
Figure 4C ACC DLD-1

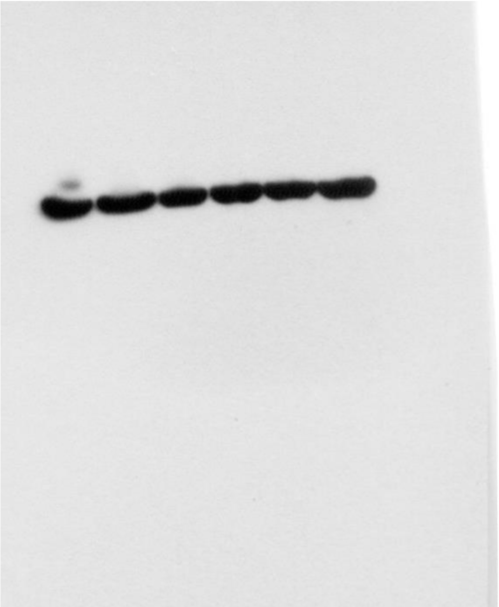
Figure 4C beta-actin DLD-1 and HCT116

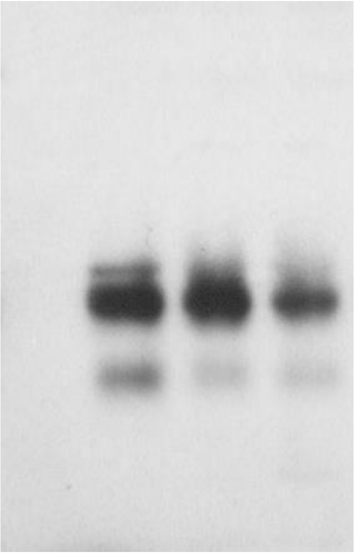

Figure 4C cMyc DLD-1

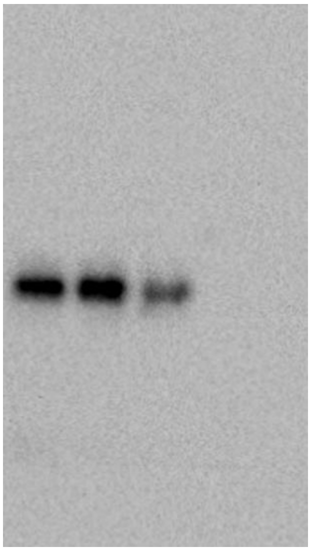

Figure 4C cMyc HCT116

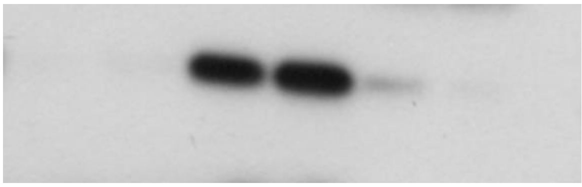

Figure 4C CPT1 DLD-1

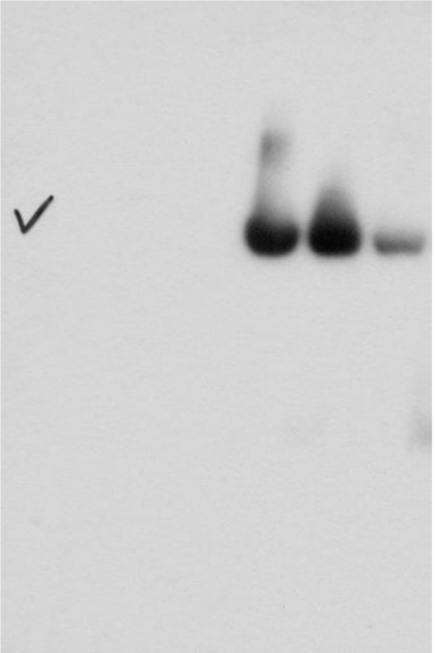

Figure 4C CPT1 HCT116

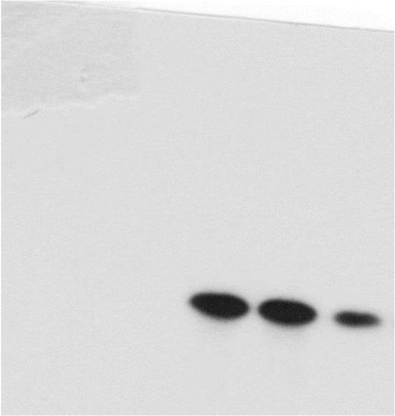

Figure 4C FASN DLD1

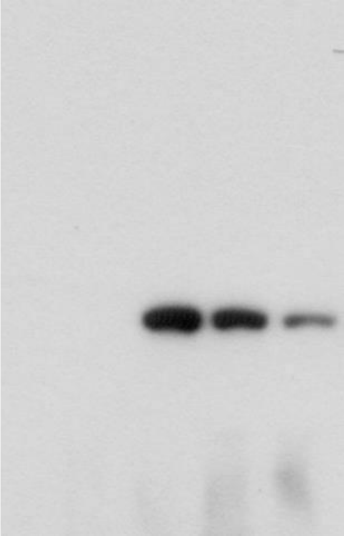

Figure 4C FASN HCT116

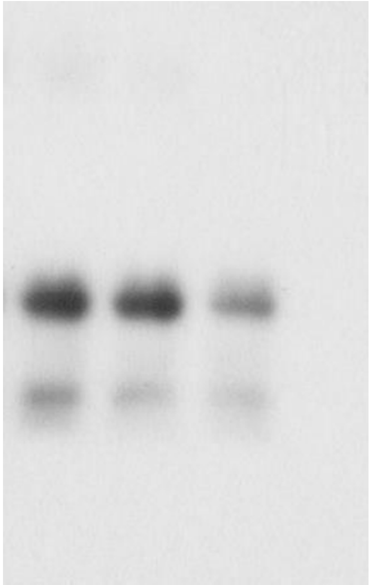

Figure 4C MCAD DLD-1

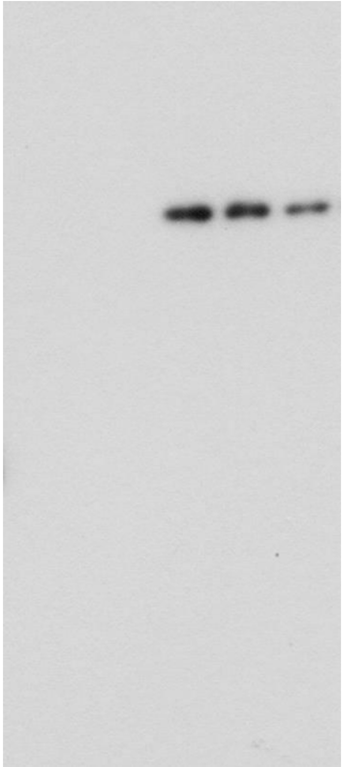

Figure 4C MCAD HCT116

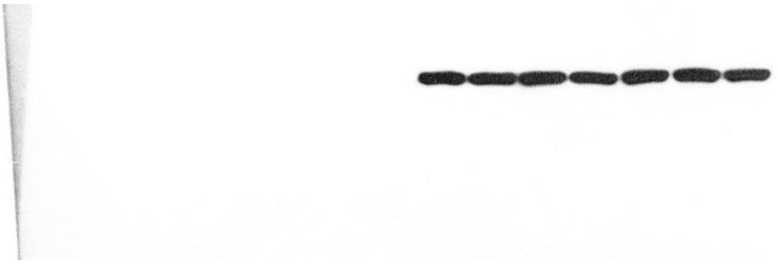

Figure 4D beta-actin

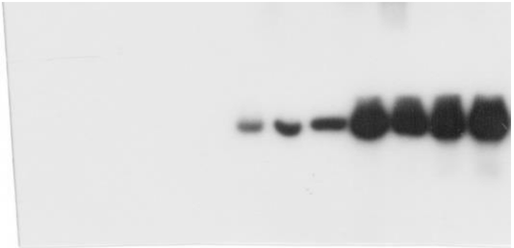

Figure 4D ACC

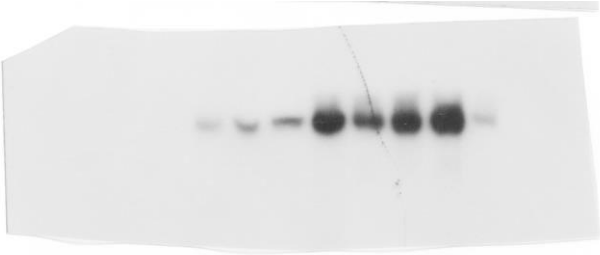

Figure 4D CPT1

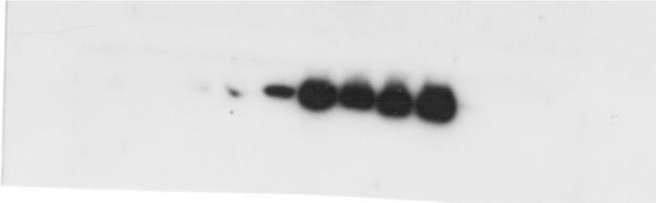

Figure 4D FASN

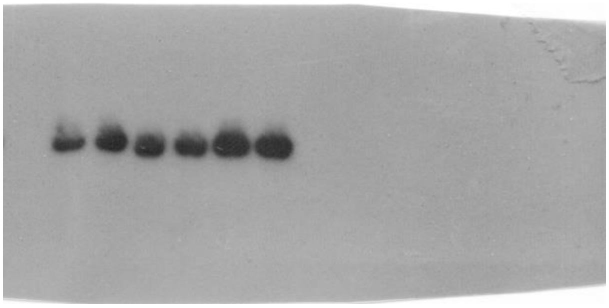

Figure 4D MCAD

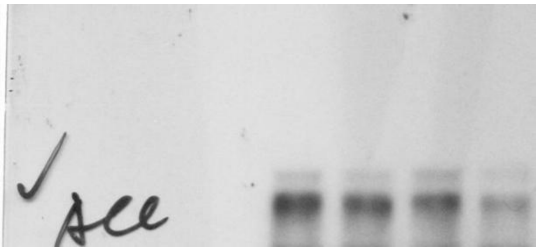

Figure 4I ACC DLD-1

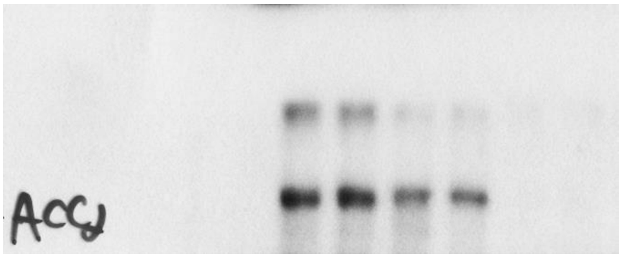

Figure 4I ACC HCT116

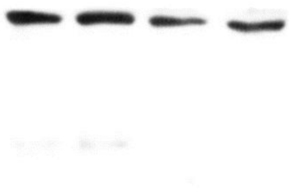

Figure 4I ACC SW480

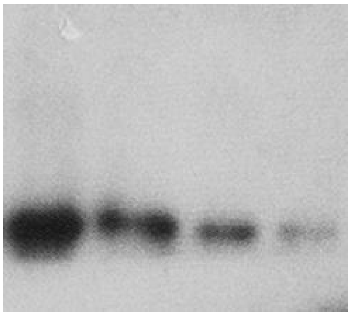

Figure 4I ACC SW620

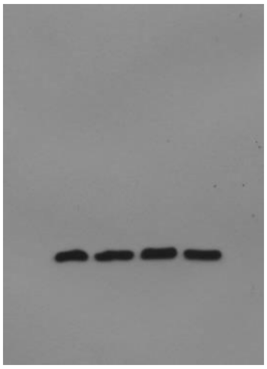

Figure 4I beta-actin DLD-1

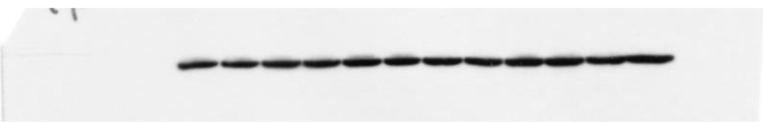

Figure 4I beta-actin SW480 SW620 HCT116

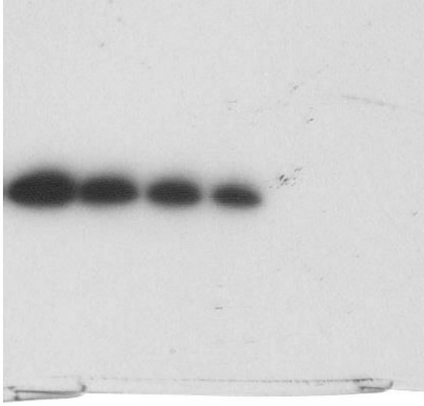

Figure 4I CPT1 DLD-1

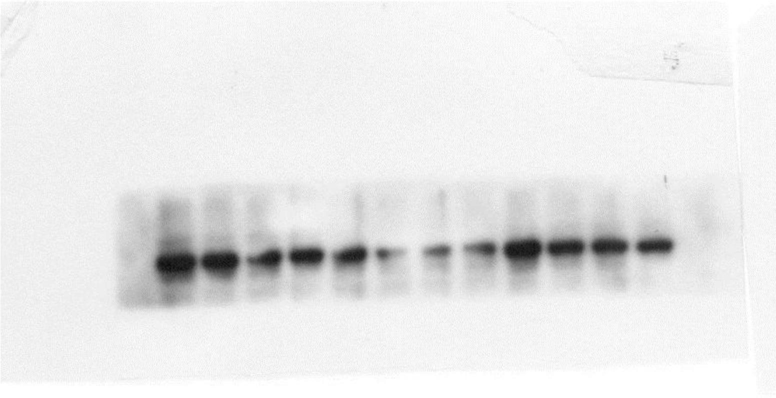

Figure 4I CPT1
SW480 SW620 HCT116

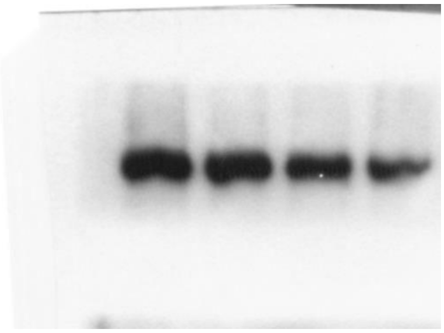

Figure 4I FASN DLD-1

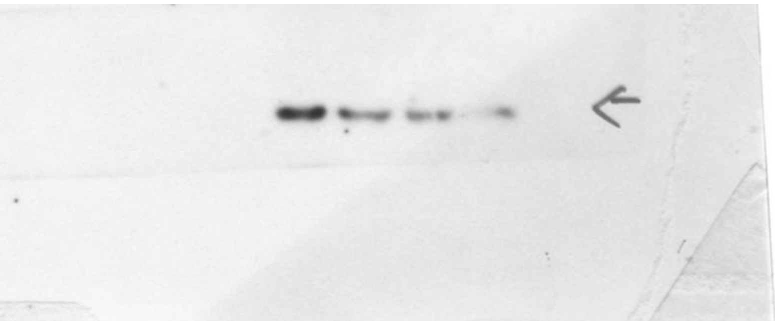

Figure 4I FASN HCT116

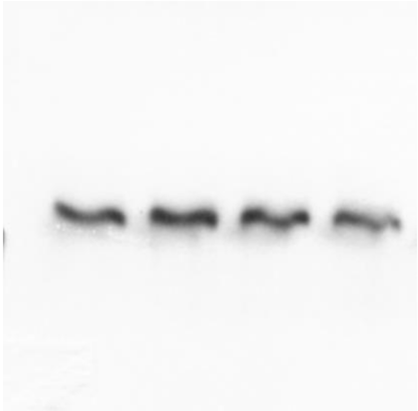

Figure 4I FASN SW480

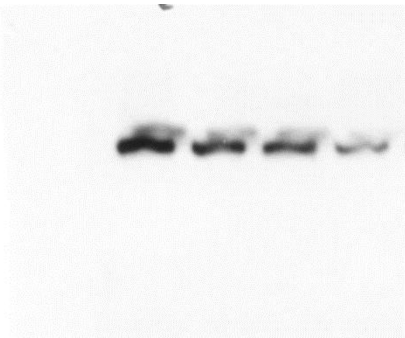

Figure 4I FASN SW620

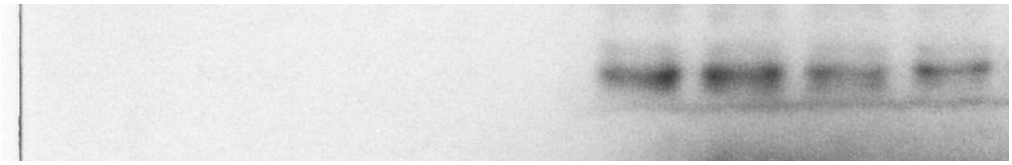

Figure 4I MCAD DLD-1

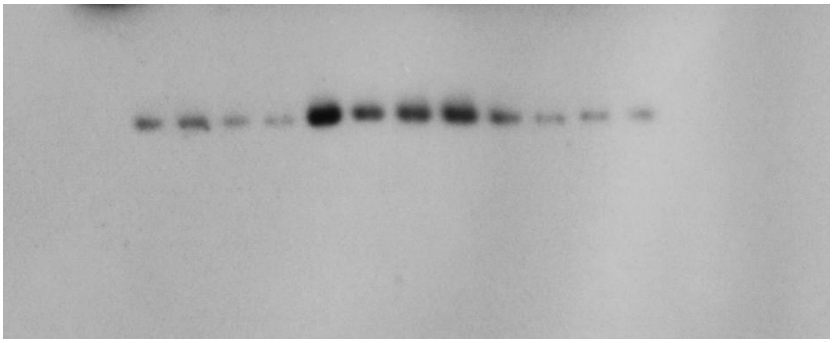

Figure 4I MCAD SW480 SW620 HCT116

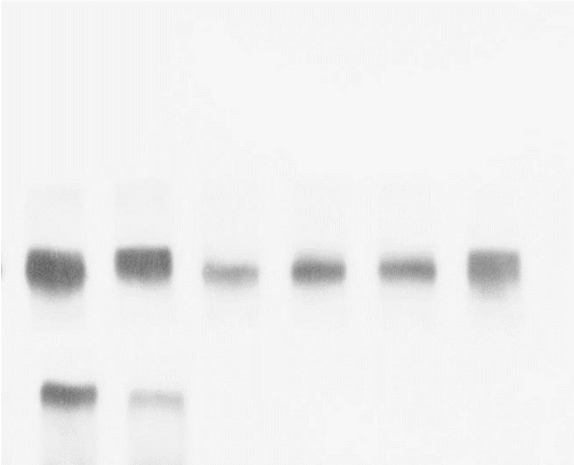

Figure 4K ACC

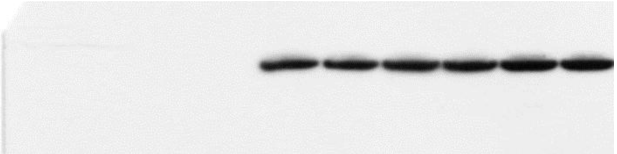

Figure 4K beta-actin

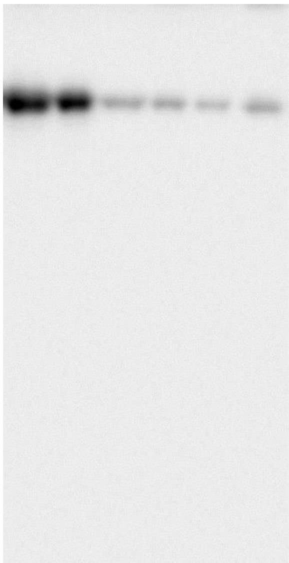

Figure 4K CPT1

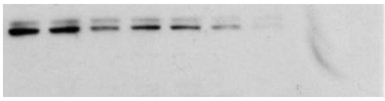

Figure 4K FASN

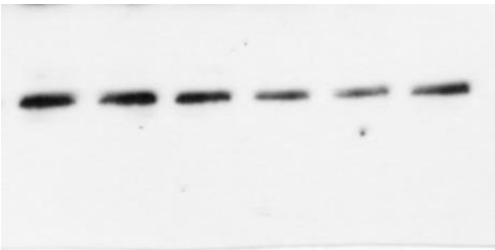

Figure 4K MCAD

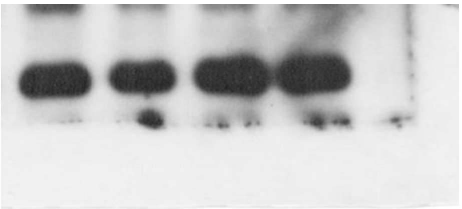

Figure 6E Bax DLD-1

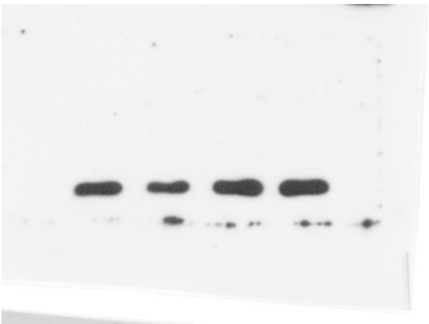

Figure 6E Bax HCT116

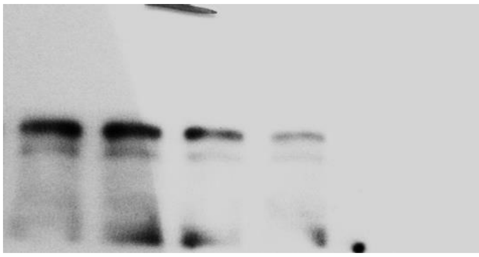

Figure 6E BCL2 DLD-1

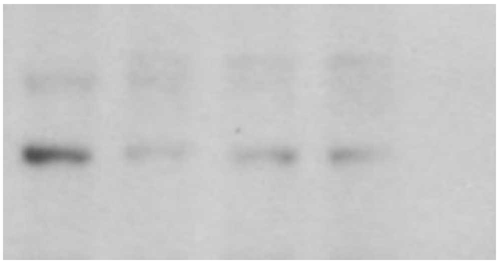

Figure 6E BCL2 HCT116

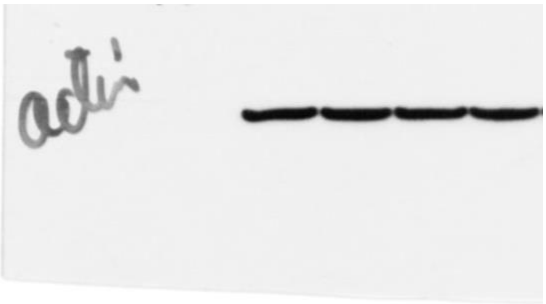

Figure 6E beta-actin DLD-1

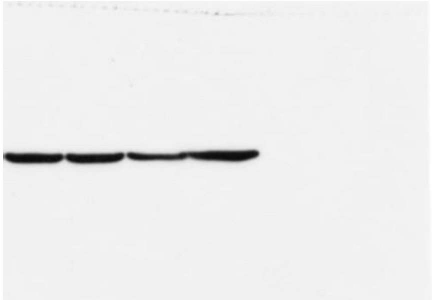

Figure 6E beta-actin HCT116

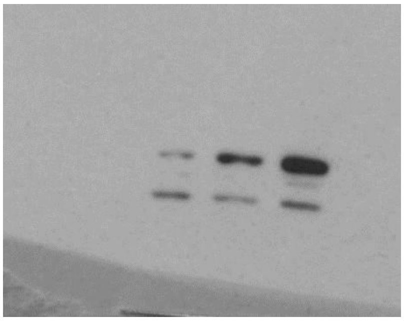

Figure 6E cleaved caspase 3 DLD-1

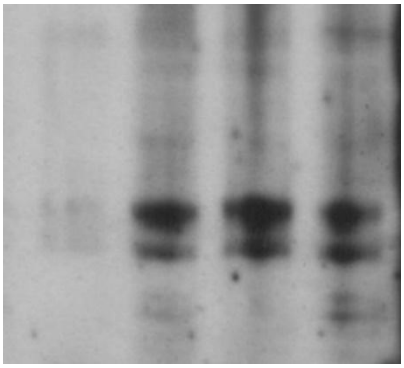

Figure 6E cleaved caspase 3 HCT116

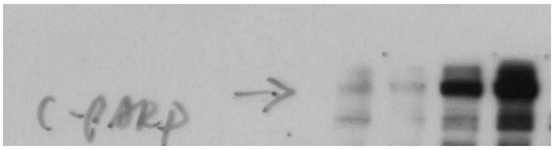

Figure 6E cleaved PARP DLD-1

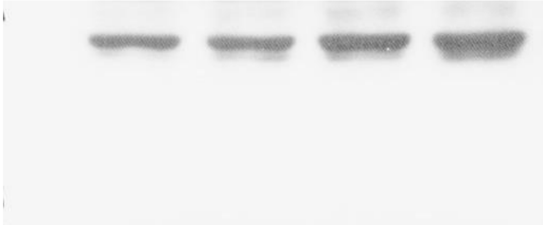

Figure 6E cleaved PARP HCT116

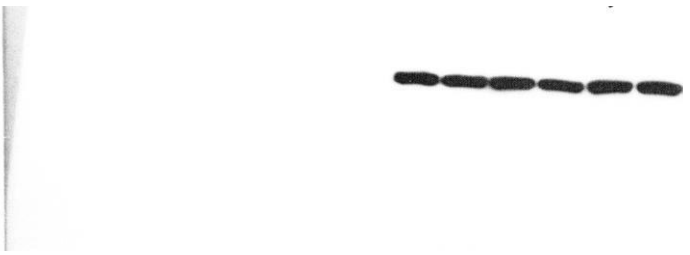

Figure 6F beta-actin

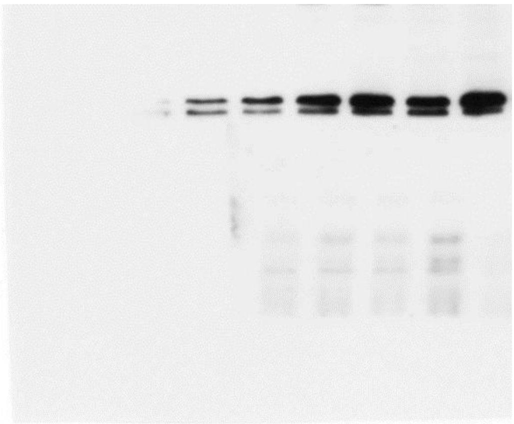

Figure 6F cleaved caspase 3

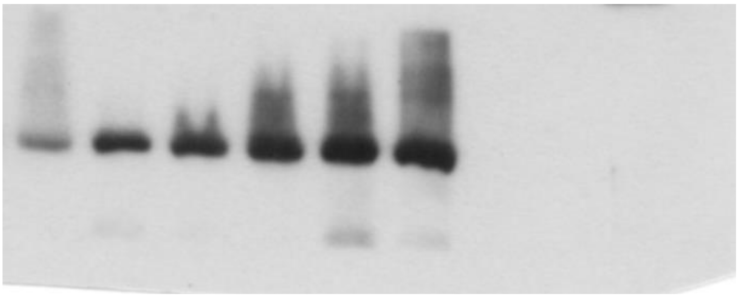

Figure 6F cleaved PARP
