# Supplementary material for: Dihydroartemisinin is potential therapeutics for treating late-stage CRC by targeting the elevated c-Myc level
Source: Cell Death Dis. 2021 Nov 5;12(11):1053. doi: 10.1038/s41419-021-04247-w (PMC8571272; doi:10.1038/s41419-021-04247-w)
Supplement: Supplementary file 4 — ccids-author-contribution form [file 41419_2021_4247_MOESM4_ESM.pdf]

DECLARATION OF CONTRIBUTIONS TO ARTICLE

ADMC

Manuscript Number: CDDIS-21-1422

Journal Name: *Cell Death & Disease* (the 'Journal')

Proposed Title of the Contribution:

Dihydroartemisinin is potential therapeutics for treating late-stage CRC by targeting the elevated c-Myc level (the 'Contribution)

Author(s):

Xianjing Hu, Sarwat Fatima, Minting Chen, Tao Huang, Yuen Wa Chen, Ruihong Gong, Hoi Leong Xavier Wong, Rongmin Yu, Liyan Song, Hiu Yee Kwan, Zhaoxiang Bian (the 'Authors')

For all *CDDis* articles, each person named as an author in the published version must be able to show he or she has contributed substantially to the article.

Authorship credit should be based on 1) substantial contributions to conception and design, acquisition of data, or analysis and interpretation of data; 2) drafting the article or revising it critically for important intellectual content; and 3) final approval of the version to be published. Authors should meet conditions 1, 2 and 3.

Any person who cannot be shown to have made a substantial contribution to the article cannot be listed as an author in the final version. The name of any person who is deemed to have made a minor contribution can, however, appear in the Acknowledgments section of the article.

Please complete the table below to indicate the contributions of all named authors to the manuscript.

Author Full Name:	Specification of Contribution to the Manuscript:
Xianjing Hu	Data curation and formal analysis, writing
Sarwat Fatima	Data curation and formal analysis, writing
Minting Chen	Data curation and formal analysis
Tao Huang	Data curation and formal analysis
Yuen Wa Chen	Data curation and formal analysis
Ruihong Gong	Data curation and formal analysis
Hoi Leong Xavier Wong	Review and editing
Rongmin Yu	Review and editing
Liyan Song	Review and editing
Hiu Yee Kwan	Conceptualization, writing, review and editing, funding acquisition
Zhaoxiang Bian	Conceptualization, review and editing, funding acquisition

Please complete the table below to indicate the contributions of all named authors to the figures.

Figure 1:

Sarwat Fatima, Xianjing Hu

Figure 2:

Xianjing Hu, Yuen Wa Chen

Figure 3:

Xianjing Hu, Minting Chen, Ruihong Gong

Figure 4:

Xianjing Hu, Sarwat Fatima

Figure 5:

Xianjing Hu, Sarwat Fatima, Minting Chen, Tao Huang

Figure 6:

Xianjing Hu

Signed for and on behalf of the Author(s):	Print Name:	Date:
	Kwan Hiu Yee	28th July 2021
