# Supplementary material for: Dihydroartemisinin is potential therapeutics for treating late-stage CRC by targeting the elevated c-Myc level
Source: Cell Death Dis. 2021 Nov 5;12(11):1053. doi: 10.1038/s41419-021-04247-w (PMC8571272; doi:10.1038/s41419-021-04247-w)
Supplement: Supplementary file 5 — Agreement on the change of authorship [file 41419_2021_4247_MOESM5_ESM.pdf]

香港浸會大學 HONG KONG BAPTIST UNIVERSITY

Hiu Yee KWAN <hykwan@hkbu.edu.hk>

Re:Fwd: CDDIS-21-1422RRRR Initial Quality Check

1 message

胡显镜 <huxj2003@163.com> Fri, Sep 17, 2021 at 2:00 PM
To: Hiu Yee KWAN <hykwan@hkbu.edu.hk>
Cc: Zhaoxiang BIAN <bzxiang@hkbu.edu.hk>, tyrm@jnu.edu.cn, tsly@jnu.edu.cn, Sarwat Fatima <sarwatfatima@yahoo.com>, Minting CHEN <18482309@life.hkbu.edu.hk>, Huang Tao <thuangsh@gmail.com>, Ruihong GONG <gongruihong@hkbu.edu.hk>, Hoi Leong Xavier WONG <xavierwong@hkbu.edu.hk>

Dear Anna，

I agree that Chen Yuen Wa is included in the revised manuscript.

Thank you very much！

Kind regards,

-Xianjing Hu

At 2021-09-17 13:40:03, "Hiu Yee KWAN" <hykwan@hkbu.edu.hk> wrote:

> Dear All,
>
> With regard to our accepted manuscript to Cell Death Dis, titled "Dihydroartemisinin is potential therapeutics for treating late-stage CRC by targeting the elevated c-Myc level", please kindly reply to this email if you agree with the following authorship (which is requested by the journal, email below) in which Chen Yuen Wa is included for her help on the supplementary data in the revised manuscript.
>
> Dr Xianjing Hu , Dr Sarwat Fatima , Ms Minting Chen , Dr Tao Huang , Ms Yuen Wa Chen , Mr Rui-Hong Gong , Dr Hoi Leong Xavier Wong , Professor Rongmin Yu , Professor Liyan Song , Dr. Kwan Hiu Yee, Professor Zhao-xiang Bian
>
> Thank you very much.

Best regards,

Anna

****

KWAN Hiu Yee, Anna, PhD

Assistant Professor

Centre for Cancer and Inflammation Research

School of Chinese Medicine

Hong Kong Baptist University

Address: Rm 412, JCSCM Bld,

7 Baptist University Rd, Kowloon, Hong Kong.

Tel: 852-3411 2016

ORCiD: https://orcid.org/0000-0002-6088-7323

---------- Forwarded message ---------
From: <cddisease@springernature.com>
Date: Thu, Sep 16, 2021 at 7:44 PM
Subject: CDDIS-21-1422RRRR Initial Quality Check
To: <hykwan@hkbu.edu.hk>

Dear Dr Kwan,

In checking in your manuscript submitted to Cell Death & Disease it has come to our attention that the following must be addressed before we can proceed.

1. Please note that final files should not have any highlighting - please do not duplicate files either. Please check your Original WB film files for this - currently there are two uploaded, one with highlights - please remove this.

2. It has come to our attention that your most recent author list differs from the one in your original submission. It has come to or attention that Yuen Wa Chen has been added since your original submission.

Please request agreement from all authors including additions and deletions, these can be collected in the following way:

Email your co-authors with the change, and ask them to reply to your email confirming that they agree to these changes. Once you have collected these replies, please combine all of the co-authors' email responses in one document and upload this file to your submission.

Your paper has been placed back in the Author Approval Folder where you may access via the following link:

https://mts-cddis.nature.com/cgi-bin/main.plex?el=A6CA5UGW7E7YTs1F7A9ftd3sCVsU5Y1T0ao8oZkifwZ

(Press/Click on the above link to be automatically sent to the web page.)

Please make the correction(s) as specified above and resubmit your paper following the same steps as before.

If you have any questions please feel free to contact us.

Sincerely,

Editorial Office
Cell Death & Disease
cddisease@springernature.com

Our flexible approach during the COVID-19 pandemic
If you need more time at any stage of the peer-review process, please do let us know. While our systems will continue to remind you of the original timelines, we aim to be as flexible as possible during the current pandemic.

This email has been sent through the NPG Manuscript Tracking System NY-610A-NPG&MTS

Confidentiality Statement:

This e-mail is confidential and subject to copyright. Any unauthorised use or disclosure of its contents is prohibited. If you have received this email in error please notify our Manuscript Tracking System Helpdesk team at http://platformsupport.nature.com .

Details of the confidentiality and pre-publicity policy may be found here http://www.nature.com/authors/policies/confidentiality.html

Privacy Policy | Update Profile

Disclaimer

This message (including any attachments) may contain confidential information intended for a specific individual and/or purpose. If you are not the intended recipient, please delete this message and notify the sender and the University immediately. Any disclosure, copying, or distribution of this message, or the taking of any action based on it, is prohibited as it may be unlawful.

In addition, the University specifically denies any responsibility for the accuracy or quality of information obtained through University E-mail Facilities. Any views and opinions expressed in the email(s) are those of the author(s), and do not necessarily represent the views and opinions of the University. The University accepts no liability whatsoever for any losses or damages that may be incurred or caused to any party as a result of the use of such information.

香港浸會大學 HONG KONG BAPTIST UNIVERSITY

Hiu Yee KWAN <hykwan@hkbu.edu.hk>

Re: CDDIS-21-1422RRRR Initial Quality Check

1 message

Sarwat Fatima <sarwatfatima@yahoo.com> Fri, Sep 17, 2021 at 2:06 PM
To: Hiu Yee KWAN <hykwan@hkbu.edu.hk>, zx Bian <bianzxiang@gmail.com>
Cc: Zhaoxiang BIAN <bzxiang@hkbu.edu.hk>, tyrm <tyrm@jnu.edu.cn>, "tsly@jnu.edu.cn" <tsly@jnu.edu.cn>, 胡显镜 <huxj2003@163.com>, Minting CHEN <18482309@life.hkbu.edu.hk>, Huang Tao <thuangsh@gmail.com>, Ruihong GONG <gongruihong@hkbu.edu.hk>, Hoi Leong Xavier WONG <xavierwong@hkbu.edu.hk>

Dear Anna,

I agree to the changes in the revised manuscript i.e. addition of Ms. Chen Yuen Wa to the author list.

Regards
Sarwat Fatima

On Friday, September 17, 2021, 01:54:54 PM GMT+8, zx Bian <bianzxiang@gmail.com> wrote:

Dear Anna, Agree. best b

--
BIAN Zhao-Xiang, MD., PhD, 卞兆祥
Tsang Shiu Tim Endowed Professor of Chinese Medicine Clinical Studies
Chair Professor in Chinese Medicine
Associate Vice President (Chinese Medicine Development)
Director, Centre for Chinese Herbal Medicine Drug Development
Director, Chinese EQUATOR Centre
Director, Clinical Division, School of Chinese Medicine
Director, Hong Kong Chinese Medicine Clinical Study Centre

Hong Kong Baptist University
Tel: (852)-34112905
Fax: (852)-34112929

Disclaimer
This email (and any attachments thereto) is intended only for the person or entity to which it is addressed and may contain confidential and/or privileged materials for specific purposes. Any disclosure, retransmission, dissemination, copying, printing or other use of, or taking any actions in reliance upon, this information by persons or entities other than the intended recipient is strictly prohibited. If you have received this email in error, please notify us by replying to this email and delete it from your system immediately. Any email and attachments thereto should be scanned to detect viruses. Neither the University nor the sender accepts any responsibility for viruses.

Hiu Yee KWAN <hykwan@hkbu.edu.hk> 於 2021年9月17日週五 下午1:40寫道：

Dear All,

With regard to our accepted manuscript to Cell Death Dis, titled "Dihydroartemisinin is potential therapeutics for treating late-stage CRC by targeting the elevated c-Myc level", please kindly reply to this email if you agree with the following authorship (which is requested by the journal, email below) in which Chen Yuen Wa is included for her help on the supplementary data in the revised manuscript.

Dr Xianjing Hu , Dr Sarwat Fatima , Ms Minting Chen , Dr Tao Huang , Ms Yuen Wa Chen , Mr Rui-Hong Gong , Dr Hoi Leong Xavier Wong , Professor Rongmin Yu , Professor Liyan Song , Dr. Kwan Hiu Yee, Professor Zhao-xiang Bian

Thank you very much.

Best regards,
Anna

****

KWAN Hiu Yee, Anna, PhD
Assistant Professor
Centre for Cancer and Inflammation Research
School of Chinese Medicine
Hong Kong Baptist University
Address: Rm 412, JCSCM Bld,
7 Baptist University Rd, Kowloon, Hong Kong.
Tel: 852-3411 2016
ORCiD: https://orcid.org/0000-0002-6088-7323

---------- Forwarded message ---------
From: <cddisease@springernature.com>
Date: Thu, Sep 16, 2021 at 7:44 PM
Subject: CDDIS-21-1422RRRR Initial Quality Check
To: <hykwan@hkbu.edu.hk>

Dear Dr Kwan,

In checking in your manuscript submitted to Cell Death & Disease it has come to our attention that the following must be addressed before we can proceed.

1. Please note that final files should not have any highlighting - please do not duplicate files either. Please check your Original WB film files for this - currently there are two uploaded, one with highlights - please remove this.

2. It has come to our attention that your most recent author list differs from the one in your original submission. It has come to or attention that Yuen Wa Chen has been added since your original submission.

Please request agreement from all authors including additions and deletions, these can be collected in the following way:

Email your co-authors with the change, and ask them to reply to your email confirming that they agree to these changes. Once you have collected these replies, please combine all of the co-authors’ email responses in one document and upload this file to your submission.

Your paper has been placed back in the Author Approval Folder where you may access via the following link:

https://mts-cddis.nature.com/cgi-bin/main.plex?el=A6CA5UGW7E7YTs1F7A9ftd3sCVsU5Y1T0ao8oZkifwZ

(Press/Click on the above link to be automatically sent to the web page.)

Please make the correction(s) as specified above and resubmit your paper following the same steps as before.

If you have any questions please feel free to contact us.

Sincerely,

Editorial Office
Cell Death & Disease
cddisease@springernature.com

Our flexible approach during the COVID-19 pandemic
If you need more time at any stage of the peer-review process, please do let us know. While our systems will continue to remind you of the original timelines, we aim to be as flexible as possible during the current pandemic.

This email has been sent through the NPG Manuscript Tracking System NY-610A-NPG&MTS

Confidentiality Statement:

This e-mail is confidential and subject to copyright. Any unauthorised use or disclosure of its contents is prohibited. If you have received this email in error please notify our Manuscript Tracking System Helpdesk team at http://platformsupport.nature.com .

Details of the confidentiality and pre-publicity policy may be found here http://www.nature.com/authors/policies/confidentiality.html

Privacy Policy | Update Profile

Disclaimer

This message (including any attachments) may contain confidential information intended for a specific individual and/or purpose. If you are not the intended recipient, please delete this message and notify the sender and the University immediately. Any disclosure, copying, or distribution of this message, or the taking of any action based on it, is prohibited as it may be unlawful.

In addition, the University specifically denies any responsibility for the accuracy or quality of information obtained through University E-mail Facilities. Any views and opinions expressed in the email(s) are those of the author(s), and do not necessarily represent the views and opinions of the University. The University accepts no liability whatsoever for any losses or damages that may be incurred or caused to any party as a result of the use of such information.

香港浸會大學 HONG KONG BAPTIST UNIVERSITY

Hiu Yee KWAN <hykwan@hkbu.edu.hk>

Re: CDDIS-21-1422RRRR Initial Quality Check

1 message

Minting CHEN <18482309@life.hkbu.edu.hk> Fri, Sep 17, 2021 at 9:39 PM
To: Hiu Yee KWAN <hykwan@hkbu.edu.hk>
Cc: Hoi Leong Xavier WONG <xavierwong@hkbu.edu.hk>, Huang Tao <thuangsh@gmail.com>, Ruihong GONG <gongruihong@hkbu.edu.hk>, Sarwat Fatima <sarwatfatima@yahoo.com>, Zhaoxiang BIAN <bzxiang@hkbu.edu.hk>, "tsly@jnu.edu.cn" <tsly@jnu.edu.cn>, tyrm <tyrm@jnu.edu.cn>, zx Bian <bianzxiang@gmail.com>, 胡显镜 <huxj2003@163.com>

Dear Dr. Kwan,

I agree with the changed authorship. Thanks a lot.

Best regards,
Minting

Huang Tao <thuangsh@gmail.com>于2021年9月17日 周五下午2:33写道：

> Dear Anna,
>
> I agree with the changed authorship.
>
> Best,
> Tao
>
> Sarwat Fatima <sarwatfatima@yahoo.com> 于2021年9月17日周五 下午2:06写道：
>
>> Dear Anna,
>>
>> I agree to the changes in the revised manuscript i.e. addition of Ms. Chen Yuen Wa to the author list.
>>
>> Regards
>> Sarwat Fatima
>>
>> On Friday, September 17, 2021, 01:54:54 PM GMT+8, zx Bian <bianzxiang@gmail.com> wrote:
>>
>> Dear Anna, Agree. best b
>>
>> --
>> BIAN Zhao-Xiang, MD., PhD, 卞兆祥
>> Tsang Shiu Tim Endowed Professor of Chinese Medicine Clinical Studies
>> Chair Professor in Chinese Medicine
>> Associate Vice President (Chinese Medicine Development)

Director, Centre for Chinese Herbal Medicine Drug Development
Director, Chinese EQUATOR Centre
Director, Clinical Division, School of Chinese Medicine
Director, Hong Kong Chinese Medicine Clinical Study Centre

Hong Kong Baptist University
Tel: (852)-34112905
Fax: (852)-34112929

Disclaimer
This email (and any attachments thereto) is intended only for the person or entity to which it is addressed and may contain confidential and/or privileged materials for specific purposes. Any disclosure, retransmission, dissemination, copying, printing or other use of, or taking any actions in reliance upon, this information by persons or entities other than the intended recipient is strictly prohibited. If you have received this email in error, please notify us by replying to this email and delete it from your system immediately. Any email and attachments thereto should be scanned to detect viruses. Neither the University nor the sender accepts any responsibility for viruses.

Hiu Yee KWAN <hykwan@hkbu.edu.hk> 於 2021年9月17日週五 下午1:40寫道：

Dear All,

With regard to our accepted manuscript to Cell Death Dis, titled "Dihydroartemisinin is potential therapeutics for treating late-stage CRC by targeting the elevated c-Myc level", please kindly reply to this email if you agree with the following authorship (which is requested by the journal, email below) in which Chen Yuen Wa is included for her help on the supplementary data in the revised manuscript.

Dr Xianjing Hu , Dr Sarwat Fatima , Ms Minting Chen , Dr Tao Huang , Ms Yuen Wa Chen , Mr Rui-Hong Gong , Dr Hoi Leong Xavier Wong , Professor Rongmin Yu , Professor Liyan Song , Dr. Kwan Hiu Yee, Professor Zhao-xiang Bian

Thank you very much.

Best regards,
Anna

****

KWAN Hiu Yee, Anna, PhD
Assistant Professor
Centre for Cancer and Inflammation Research
School of Chinese Medicine
Hong Kong Baptist University
Address: Rm 412, JCSCM Bld,
7 Baptist University Rd, Kowloon, Hong Kong.
Tel: 852-3411 2016
ORCiD: https://orcid.org/0000-0002-6088-7323

---------- Forwarded message ---------
From: <cddisease@springernature.com>
Date: Thu, Sep 16, 2021 at 7:44 PM
Subject: CDDIS-21-1422RRRR Initial Quality Check
To: <hykwan@hkbu.edu.hk>

Dear Dr Kwan,

In checking in your manuscript submitted to Cell Death & Disease it has come to our attention that the following must be addressed before we can proceed.

1. Please note that final files should not have any highlighting - please do not duplicate files either. Please check your Original WB film files for this - currently there are two uploaded, one with highlights - please remove this.

2. It has come to our attention that your most recent author list differs from the one in your original submission. It has come to or attention that Yuen Wa Chen has been added since your original submission.

Please request agreement from all authors including additions and deletions, these can be collected in the following way:

Email your co-authors with the change, and ask them to reply to your email confirming that they agree to these changes. Once you have collected these replies, please combine all of the co-authors' email responses in one document and upload this file to your submission.

Your paper has been placed back in the Author Approval Folder where you may access via the following link:

https://mts-cddis.nature.com/cgi-bin/main.plex?el=A6CA5UGW7E7YTs1F7A9ftd3sCVsU5Y1T0ao8oZkifwZ

(Press/Click on the above link to be automatically sent to the web page.)

Please make the correction(s) as specified above and resubmit your paper following the same steps as before.

If you have any questions please feel free to contact us.

Sincerely,

Editorial Office
Cell Death & Disease
cddisease@springernature.com

Our flexible approach during the COVID-19 pandemic
If you need more time at any stage of the peer-review process, please do let us know. While our systems will continue to remind you of the original timelines, we aim to be as flexible as possible during the current pandemic.

This email has been sent through the NPG Manuscript Tracking System NY-610A-NPG&MTS

Confidentiality Statement:

This e-mail is confidential and subject to copyright. Any unauthorised use or disclosure of its contents is prohibited. If you have received this email in error please notify our Manuscript Tracking System Helpdesk team at http://platformsupport.nature.com .

Details of the confidentiality and pre-publicity policy may be found here http://www.nature.com/authors/policies/confidentiality.html

Privacy Policy | Update Profile

Disclaimer

This message (including any attachments) may contain confidential information intended for a specific individual and/or purpose. If you are not the intended recipient, please delete this message and notify the sender and the University immediately. Any disclosure, copying, or distribution of this message, or the taking of any action based on it, is prohibited as it may be unlawful.

In addition, the University specifically denies any responsibility for the accuracy or quality of information obtained through University E-mail Facilities. Any views and opinions expressed in the email(s) are those of the author(s), and do not necessarily represent the views and opinions of the University. The University accepts no liability whatsoever for any losses or damages that may be incurred or caused to any party as a result of the use of such information.

香港浸會大學
HONG KONG BAPTIST UNIVERSITY

Hiu Yee KWAN <hykwan@hkbu.edu.hk>

Re: CDDIS-21-1422RRRR Initial Quality Check

1 message

Huang Tao <thuangsh@gmail.com> Fri, Sep 17, 2021 at 2:33 PM
To: Sarwat Fatima <sarwatfatima@yahoo.com>
Cc: Hiu Yee KWAN <hykwan@hkbu.edu.hk>, zx Bian <bianzxiang@gmail.com>, Zhaoxiang BIAN <bzxiang@hkbu.edu.hk>, tyrm <tyrm@jnu.edu.cn>, "tsly@jnu.edu.cn" <tsly@jnu.edu.cn>, 胡显镜 <huxj2003@163.com>, Minting CHEN <18482309@life.hkbu.edu.hk>, Ruihong GONG <gongruihong@hkbu.edu.hk>, Hoi Leong Xavier WONG <xavierwong@hkbu.edu.hk>

Dear Anna,

I agree with the changed authorship.

Best,
Tao

Sarwat Fatima <sarwatfatima@yahoo.com> 于2021年9月17日周五 下午2:06写道：

> Dear Anna,
>
> I agree to the changes in the revised manuscript i.e. addition of Ms. Chen Yuen Wa to the author list.
>
> Regards
> Sarwat Fatima
>
> On Friday, September 17, 2021, 01:54:54 PM GMT+8, zx Bian <bianzxiang@gmail.com> wrote:
>
> Dear Anna, Agree. best b
>
> --
> BIAN Zhao-Xiang, MD., PhD, 卞兆祥
> Tsang Shiu Tim Endowed Professor of Chinese Medicine Clinical Studies
> Chair Professor in Chinese Medicine
> Associate Vice President (Chinese Medicine Development)
> Director, Centre for Chinese Herbal Medicine Drug Development
> Director, Chinese EQUATOR Centre
> Director, Clinical Division, School of Chinese Medicine
> Director, Hong Kong Chinese Medicine Clinical Study Centre
>
> Hong Kong Baptist University
> Tel: (852)-34112905
> Fax: (852)-34112929

Disclaimer
This email (and any attachments thereto) is intended only for the person or entity to which it is addressed and may contain confidential and/or privileged materials for specific purposes. Any disclosure, retransmission, dissemination, copying, printing or other use of, or taking any actions in reliance upon, this information by persons or entities other than the intended recipient is strictly prohibited. If you have received this email in error, please notify us by replying to this email and delete it from your system immediately. Any email and attachments thereto should be scanned to detect viruses. Neither the University nor the sender accepts any responsibility for viruses.

Hiu Yee KWAN <hykwan@hkbu.edu.hk> 於 2021年9月17日週五 下午1:40寫道：

Dear All,

With regard to our accepted manuscript to Cell Death Dis, titled "Dihydroartemisinin is potential therapeutics for treating late-stage CRC by targeting the elevated c-Myc level", please kindly reply to this email if you agree with the following authorship (which is requested by the journal, email below) in which Chen Yuen Wa is included for her help on the supplementary data in the revised manuscript.

Dr Xianjing Hu , Dr Sarwat Fatima , Ms Minting Chen , Dr Tao Huang , Ms Yuen Wa Chen , Mr Rui-Hong Gong , Dr Hoi Leong Xavier Wong , Professor Rongmin Yu , Professor Liyan Song , Dr. Kwan Hiu Yee, Professor Zhao-xiang Bian

Thank you very much.

Best regards,
Anna

****
KWAN Hiu Yee, Anna, PhD
Assistant Professor
Centre for Cancer and Inflammation Research
School of Chinese Medicine
Hong Kong Baptist University
Address: Rm 412, JCSCM Bld,
7 Baptist University Rd, Kowloon, Hong Kong.
Tel: 852-3411 2016
ORCiD: https://orcid.org/0000-0002-6088-7323

---------- Forwarded message ---------
From: <cddisease@springernature.com>
Date: Thu, Sep 16, 2021 at 7:44 PM
Subject: CDDIS-21-1422RRRR Initial Quality Check
To: <hykwan@hkbu.edu.hk>

Dear Dr Kwan,

In checking in your manuscript submitted to Cell Death & Disease it has come to our attention that the following must be addressed before we can proceed.

1. Please note that final files should not have any highlighting - please do not duplicate files either. Please check your Original WB film files for this - currently there are two uploaded, one with highlights - please remove this.

2. It has come to our attention that your most recent author list differs from the one in your original submission. It has come to or attention that Yuen Wa Chen has been added since your original submission.

Please request agreement from all authors including additions and deletions, these can be collected in the following way:

Email your co-authors with the change, and ask them to reply to your email confirming that they agree to these changes. Once you have collected these replies, please combine all of the co-authors' email responses in one document and upload this file to your submission.

Your paper has been placed back in the Author Approval Folder where you may access via the following link:

https://mts-cddis.nature.com/cgi-bin/main.plex?el=A6CA5UGW7E7YTs1F7A9ftd3sCVsU5Y1T0ao8oZkifwZ

(Press/Click on the above link to be automatically sent to the web page.)

Please make the correction(s) as specified above and resubmit your paper following the same steps as before.

If you have any questions please feel free to contact us.

Sincerely,

Editorial Office
Cell Death & Disease
cddisease@springernature.com

Our flexible approach during the COVID-19 pandemic
If you need more time at any stage of the peer-review process, please do let us know. While our systems will continue to remind you of the original timelines, we aim to be as flexible as possible during the current pandemic.

This email has been sent through the NPG Manuscript Tracking System NY-610A-NPG&MTS

Confidentiality Statement:

This e-mail is confidential and subject to copyright. Any unauthorised use or disclosure of its contents is prohibited. If you have received this email in error please notify our Manuscript Tracking System Helpdesk team at http://platformsupport.nature.com .

Details of the confidentiality and pre-publicity policy may be found here http://www.nature.com/authors/policies/confidentiality.html

Privacy Policy | Update Profile

Disclaimer

This message (including any attachments) may contain confidential information intended for a specific individual and/or purpose. If you are not the intended recipient, please delete this message and notify the sender and the University immediately. Any disclosure, copying, or distribution of this message, or the taking of any action based on it, is prohibited as it may be unlawful.

In addition, the University specifically denies any responsibility for the accuracy or quality of information obtained through University E-mail Facilities. Any views and opinions expressed in the email(s) are those of the author(s), and do not necessarily represent the views and opinions of the University. The University accepts no liability whatsoever for any losses or damages that may be incurred or caused to any party as a result of the use of such information.

香港浸會大學
HONG KONG BAPTIST UNIVERSITY

Hiu Yee KWAN <hykwan@hkbu.edu.hk>

Re: CDDIS-21-1422RRRR Initial Quality Check

1 message

c heidi <cywheidi@gmail.com> Fri, Sep 17, 2021 at 10:06 PM
To: Hiu Yee KWAN <hykwan@hkbu.edu.hk>

Dear Dr. Kwan,

I agree with the changed authorship.Thank you.

Best,
Chen yuen wa

「Hiu Yee KWAN <hykwan@hkbu.edu.hk>」在 2021年9月17日週五，下午9:31 寫道：

Dear Heidi,

With regard to our accepted manuscript to Cell Death Dis, titled "Dihydroartemisinin is potential therapeutics for treating late-stage CRC by targeting the elevated c-Myc level", please kindly reply to this email if you agree with the following authorship (which is requested by the journal, email below).

Dr Xianjing Hu , Dr Sarwat Fatima , Ms Minting Chen , Dr Tao Huang , Ms Yuen Wa Chen , Mr Rui-Hong Gong , Dr Hoi Leong Xavier Wong , Professor Rongmin Yu , Professor Liyan Song , Dr. Kwan Hiu Yee, Professor Zhao-xiang Bian

Thank you very much.

Best regards,
Anna

****

KWAN Hiu Yee, Anna, PhD
Assistant Professor
Centre for Cancer and Inflammation Research
School of Chinese Medicine
Hong Kong Baptist University
Address: Rm 412, JCSCM Bld,
7 Baptist University Rd, Kowloon, Hong Kong.
Tel: 852-3411 2016
ORCiD: https://orcid.org/0000-0002-6088-7323

---------- Forwarded message ---------
From: <cddisease@springernature.com>
Date: Thu, Sep 16, 2021 at 7:44 PM
Subject: CDDIS-21-1422RRRR Initial Quality Check
To: <hykwan@hkbu.edu.hk>

Dear Dr Kwan,

In checking in your manuscript submitted to Cell Death & Disease it has come to our attention that the following must be addressed before we can proceed.

1. Please note that final files should not have any highlighting - please do not duplicate files either. Please check your Original WB film files for this - currently there are two uploaded, one with highlights - please remove this.

2. It has come to our attention that your most recent author list differs from the one in your original submission. It has come to or attention that Yuen Wa Chen has been added since your original submission.

Please request agreement from all authors including additions and deletions, these can be collected in the following way:

Email your co-authors with the change, and ask them to reply to your email confirming that they agree to these changes. Once you have collected these replies, please combine all of the co-authors' email responses in one document and upload this file to your submission.

Your paper has been placed back in the Author Approval Folder where you may access via the following link:

https://mts-cddis.nature.com/cgi-bin/main.plex?el=A6CA5UGW7E7YTs1F7A9ftd3sCVsU5Y1T0ao8oZkifwZ

(Press/Click on the above link to be automatically sent to the web page.)

Please make the correction(s) as specified above and resubmit your paper following the same steps as before.

If you have any questions please feel free to contact us.

Sincerely,

Editorial Office
Cell Death & Disease
cddisease@springernature.com

Our flexible approach during the COVID-19 pandemic
If you need more time at any stage of the peer-review process, please do let us know. While our systems will continue to remind you of the original timelines, we aim to be as flexible as possible during the current pandemic.

This email has been sent through the NPG Manuscript Tracking System NY-610A-NPG&MTS

Confidentiality Statement:

This e-mail is confidential and subject to copyright. Any unauthorised use or disclosure of its contents is prohibited. If you have received this email in error please notify our Manuscript Tracking System Helpdesk team at http://platformsupport.nature.com .

Details of the confidentiality and pre-publicity policy may be found here http://www.nature.com/authors/policies/confidentiality.html

Privacy Policy | Update Profile

Disclaimer

This message (including any attachments) may contain confidential information intended for a specific individual and/or purpose. If you are not the intended recipient, please delete this message and notify the sender and the University immediately. Any disclosure, copying, or distribution of this message, or the taking of any action based on it, is prohibited as it may be unlawful.

In addition, the University specifically denies any responsibility for the accuracy or quality of information obtained through University E-mail Facilities. Any views and opinions expressed in the email(s) are those of the author(s), and do not necessarily represent the views and opinions of the University. The University accepts no liability whatsoever for any losses or damages that may be incurred or caused to any party as a result of the use of such information.

香港浸會大學 HONG KONG BAPTIST UNIVERSITY

Hiu Yee KWAN <hykwan@hkbu.edu.hk>

Re: CDDIS-21-1422RRRR Initial Quality Check

1 message

Ruihong GONG <gongruihong@hkbu.edu.hk> Fri, Sep 17, 2021 at 1:49 PM
To: Hiu Yee KWAN <hykwan@hkbu.edu.hk>

Dear Dr Kwan,

I agree with these changes. Thank you.

Best regards,
Mr Rui-Hong Gong

On Fri, Sep 17, 2021 at 1:40 PM Hiu Yee KWAN <hykwan@hkbu.edu.hk> wrote:

Dear All,

With regard to our accepted manuscript to Cell Death Dis, titled "Dihydroartemisinin is potential therapeutics for treating late-stage CRC by targeting the elevated c-Myc level", please kindly reply to this email if you agree with the following authorship (which is requested by the journal, email below) in which Chen Yuen Wa is included for her help on the supplementary data in the revised manuscript.

Dr Xianjing Hu , Dr Sarwat Fatima , Ms Minting Chen , Dr Tao Huang , Ms Yuen Wa Chen , Mr Rui-Hong Gong , Dr Hoi Leong Xavier Wong , Professor Rongmin Yu , Professor Liyan Song , Dr. Kwan Hiu Yee, Professor Zhao-xiang Bian

Thank you very much.

Best regards,
Anna
****

KWAN Hiu Yee, Anna, PhD
Assistant Professor
Centre for Cancer and Inflammation Research
School of Chinese Medicine
Hong Kong Baptist University
Address: Rm 412, JCSCM Bld,
7 Baptist University Rd, Kowloon, Hong Kong.
Tel: 852-3411 2016
ORCiD: https://orcid.org/0000-0002-6088-7323

---------- Forwarded message ---------
From: <cddisease@springernature.com>
Date: Thu, Sep 16, 2021 at 7:44 PM

Subject: CDDIS-21-1422RRRR Initial Quality Check
To: <hykwan@hkbu.edu.hk>

Dear Dr Kwan,

In checking in your manuscript submitted to Cell Death & Disease it has come to our attention that the following must be addressed before we can proceed.

1. Please note that final files should not have any highlighting - please do not duplicate files either. Please check your Original WB film files for this - currently there are two uploaded, one with highlights - please remove this.

2. It has come to our attention that your most recent author list differs from the one in your original submission. It has come to or attention that Yuen Wa Chen has been added since your original submission.

Please request agreement from all authors including additions and deletions, these can be collected in the following way:

Email your co-authors with the change, and ask them to reply to your email confirming that they agree to these changes. Once you have collected these replies, please combine all of the co-authors' email responses in one document and upload this file to your submission.

Your paper has been placed back in the Author Approval Folder where you may access via the following link:

https://mts-cddis.nature.com/cgi-bin/main.plex?el=A6CA5UGW7E7YTs1F7A9ftd3sCVsU5Y1T0ao8oZkifwZ

(Press/Click on the above link to be automatically sent to the web page.)

Please make the correction(s) as specified above and resubmit your paper following the same steps as before.

If you have any questions please feel free to contact us.

Sincerely,

Editorial Office
Cell Death & Disease
cddisease@springernature.com

Our flexible approach during the COVID-19 pandemic
If you need more time at any stage of the peer-review process, please do let us know. While our systems will continue to remind you of the original timelines, we aim to be as flexible as possible during the current pandemic.

This email has been sent through the NPG Manuscript Tracking System NY-610A-NPG&MTS

Confidentiality Statement:

This e-mail is confidential and subject to copyright. Any unauthorised use or disclosure of its contents is prohibited. If you have received this email in error please notify our Manuscript Tracking System Helpdesk team at http://platformsupport.nature.com .

Details of the confidentiality and pre-publicity policy may be found here http://www.nature.com/authors/policies/confidentiality.html

Privacy Policy | Update Profile

香港浸會大學
HONG KONG BAPTIST UNIVERSITY

Hiu Yee KWAN <hykwan@hkbu.edu.hk>

Re: CDDIS-21-1422RRRR Initial Quality Check

1 message

Hoi Leong Xavier WONG <xavierwong@hkbu.edu.hk> Fri, Sep 17, 2021 at 2:01 PM
To: Hiu Yee KWAN <hykwan@hkbu.edu.hk>
Cc: Huang Tao <thuangsh@gmail.com>, Minting CHEN <18482309@life.hkbu.edu.hk>, Ruihong GONG <gongruihong@hkbu.edu.hk>, Sarwat Fatima <sarwatfatima@yahoo.com>, Zhaoxiang BIAN <bzxiang@hkbu.edu.hk>, tsly@jnu.edu.cn, tyrm@jnu.edu.cn, 胡显镜 <huxj2003@163.com>

Dear Anna,

I agree with the authorship. Thanks

Best,
Xavier

「Hiu Yee KWAN <hykwan@hkbu.edu.hk>」在 2021年9月17日週五，13:40 寫道：

> Dear All,
>
> With regard to our accepted manuscript to Cell Death Dis, titled "Dihydroartemisinin is potential therapeutics for treating late-stage CRC by targeting the elevated c-Myc level", please kindly reply to this email if you agree with the following authorship (which is requested by the journal, email below) in which Chen Yuen Wa is included for her help on the supplementary data in the revised manuscript.
>
> Dr Xianjing Hu , Dr Sarwat Fatima , Ms Minting Chen , Dr Tao Huang , Ms Yuen Wa Chen , Mr Rui-Hong Gong , Dr Hoi Leong Xavier Wong , Professor Rongmin Yu , Professor Liyan Song , Dr. Kwan Hiu Yee, Professor Zhao-xiang Bian
>
> Thank you very much.
>
> Best regards,
> Anna
> **

> **KWAN Hiu Yee**, Anna, PhD
> Assistant Professor
> Centre for Cancer and Inflammation Research
> School of Chinese Medicine
> Hong Kong Baptist University
> Address: Rm 412, JCSCM Bld,
> 7 Baptist University Rd, Kowloon, Hong Kong.
> **Tel:** 852-3411 2016
> **ORCiD:** https://orcid.org/0000-0002-6088-7323

---------- Forwarded message ---------
From: <cddisease@springernature.com>
Date: Thu, Sep 16, 2021 at 7:44 PM
Subject: CDDIS-21-1422RRRR Initial Quality Check
To: <hykwan@hkbu.edu.hk>

Dear Dr Kwan,

In checking in your manuscript submitted to Cell Death & Disease it has come to our attention that the following must be addressed before we can proceed.

1. Please note that final files should not have any highlighting - please do not duplicate files either. Please check your Original WB film files for this - currently there are two uploaded, one with highlights - please remove this.

2. It has come to our attention that your most recent author list differs from the one in your original submission. It has come to or attention that Yuen Wa Chen has been added since your original submission.

Please request agreement from all authors including additions and deletions, these can be collected in the following way:

Email your co-authors with the change, and ask them to reply to your email confirming that they agree to these changes. Once you have collected these replies, please combine all of the co-authors' email responses in one document and upload this file to your submission.

Your paper has been placed back in the Author Approval Folder where you may access via the following link:

https://mts-cddis.nature.com/cgi-bin/main.plex?el=A6CA5UGW7E7YTs1F7A9ftd3sCVsU5Y1T0ao8oZkifwZ

(Press/Click on the above link to be automatically sent to the web page.)

Please make the correction(s) as specified above and resubmit your paper following the same steps as before.

If you have any questions please feel free to contact us.

Sincerely,

Editorial Office
Cell Death & Disease
cddisease@springernature.com

Our flexible approach during the COVID-19 pandemic
If you need more time at any stage of the peer-review process, please do let us know. While our systems will continue to remind you of the original timelines, we aim to be as flexible as possible during the current pandemic.

This email has been sent through the NPG Manuscript Tracking System NY-610A-NPG&MTS

Confidentiality Statement:

This e-mail is confidential and subject to copyright. Any unauthorised use or disclosure of its contents is prohibited. If you have received this email in error please notify our Manuscript Tracking System Helpdesk team at http://platformsupport.nature.com .

Details of the confidentiality and pre-publicity policy may be found here http://www.nature.com/authors/policies/confidentiality.html

Privacy Policy | Update Profile

--

WONG Hoi Leong Xavier, PhD
Assistant Professor
Teaching and Research Division
School of Chinese Medicine
Hong Kong Baptist University
Tel: 3411-2805
email: xavierwong@hkbu.edu.hk

香港浸會大學 HONG KONG BAPTIST UNIVERSITY

Hiu Yee KWAN <hykwan@hkbu.edu.hk>

(no subject)

1 message

tyrm <tyrm@jnu.edu.cn> Sat, Sep 18, 2021 at 4:12 PM
To: hykwan@hkbu.edu.hk
Cc: 胡显镜 <huxj2003@163.com>

Dear Anna,

I agree with the changed authorship.

Best wishes.

Rongmin Yu

香港浸會大學
HONG KONG BAPTIST UNIVERSITY

Hiu Yee KWAN <hykwan@hkbu.edu.hk>

I agree with the changed authorship. Thank you.

1 message

tsly <tsly@jnu.edu.cn> Sat, Sep 18, 2021 at 7:43 PM
To: hykwan@hkbu.edu.hk

Dear Anna,

Thank you for your email. I agree the changed authorship.

Best regards,

Liyan Song

香港浸會大學 HONG KONG BAPTIST UNIVERSITY

Hiu Yee KWAN <hykwan@hkbu.edu.hk>

Re: CDDIS-21-1422RRRR Initial Quality Check

1 message

zx Bian <bianzxiang@gmail.com> Fri, Sep 17, 2021 at 1:54 PM
To: Hiu Yee KWAN <hykwan@hkbu.edu.hk>
Cc: Zhaoxiang BIAN <bzxiang@hkbu.edu.hk>, tyrm <tyrm@jnu.edu.cn>, tsly@jnu.edu.cn, 胡显镜 <huxj2003@163.com>, Sarwat Fatima <sarwatfatima@yahoo.com>, Minting CHEN <18482309@life.hkbu.edu.hk>, Huang Tao <thuangsh@gmail.com>, Ruihong GONG <gongruihong@hkbu.edu.hk>, Hoi Leong Xavier WONG <xavierwong@hkbu.edu.hk>

Dear Anna, Agree. best b

--

BIAN Zhao-Xiang, MD., PhD, 卞兆祥
Tsang Shiu Tim Endowed Professor of Chinese Medicine Clinical Studies
Chair Professor in Chinese Medicine
Associate Vice President (Chinese Medicine Development)
Director, Centre for Chinese Herbal Medicine Drug Development
Director, Chinese EQUATOR Centre
Director, Clinical Division, School of Chinese Medicine
Director, Hong Kong Chinese Medicine Clinical Study Centre

Hong Kong Baptist University
Tel: (852)-34112905
Fax: (852)-34112929

Disclaimer
This email (and any attachments thereto) is intended only for the person or entity to which it is addressed and may contain confidential and/or privileged materials for specific purposes. Any disclosure, retransmission, dissemination, copying, printing or other use of, or taking any actions in reliance upon, this information by persons or entities other than the intended recipient is strictly prohibited. If you have received this email in error, please notify us by replying to this email and delete it from your system immediately. Any email and attachments thereto should be scanned to detect viruses. Neither the University nor the sender accepts any responsibility for viruses.

Hiu Yee KWAN <hykwan@hkbu.edu.hk> 於 2021年9月17日週五 下午1:40寫道：

> Dear All,
>
> With regard to our accepted manuscript to Cell Death Dis, titled "Dihydroartemisinin is potential therapeutics for treating late-stage CRC by targeting the elevated c-Myc level", please kindly reply to this email if you agree with the following authorship (which is requested by the journal, email below) in which Chen Yuen Wa is included for her help on the supplementary data in the revised manuscript.
>
> Dr Xianjing Hu , Dr Sarwat Fatima , Ms Minting Chen , Dr Tao Huang , Ms Yuen Wa Chen , Mr Rui-Hong Gong , Dr Hoi Leong Xavier Wong , Professor Rongmin Yu , Professor Liyan Song , Dr. Kwan Hiu Yee, Professor Zhao-xiang Bian

Thank you very much.

Best regards,
Anna
****

KWAN Hiu Yee, Anna, PhD
Assistant Professor
Centre for Cancer and Inflammation Research
School of Chinese Medicine
Hong Kong Baptist University
Address: Rm 412, JCSCM Bld,
7 Baptist University Rd, Kowloon, Hong Kong.
Tel: 852-3411 2016
ORCiD: https://orcid.org/0000-0002-6088-7323

---------- Forwarded message ---------
From: <cddisease@springernature.com>
Date: Thu, Sep 16, 2021 at 7:44 PM
Subject: CDDIS-21-1422RRRR Initial Quality Check
To: <hykwan@hkbu.edu.hk>

Dear Dr Kwan,

In checking in your manuscript submitted to Cell Death & Disease it has come to our attention that the following must be addressed before we can proceed.

1. Please note that final files should not have any highlighting - please do not duplicate files either. Please check your Original WB film files for this - currently there are two uploaded, one with highlights - please remove this.

2. It has come to our attention that your most recent author list differs from the one in your original submission. It has come to or attention that Yuen Wa Chen has been added since your original submission.

Please request agreement from all authors including additions and deletions, these can be collected in the following way:

Email your co-authors with the change, and ask them to reply to your email confirming that they agree to these changes. Once you have collected these replies, please combine all of the co-authors' email responses in one document and upload this file to your submission.

Your paper has been placed back in the Author Approval Folder where you may access via the following link:

https://mts-cddis.nature.com/cgi-bin/main.plex?el=A6CA5UGW7E7YTs1F7A9ftd3sCVsU5Y1T0ao8oZkifwZ

(Press/Click on the above link to be automatically sent to the web page.)

Please make the correction(s) as specified above and resubmit your paper following the same steps as before.

If you have any questions please feel free to contact us.

Sincerely,

Editorial Office
Cell Death & Disease
cddisease@springernature.com

Our flexible approach during the COVID-19 pandemic
If you need more time at any stage of the peer-review process, please do let us know. While our systems will continue to remind you of the original timelines, we aim to be as flexible as possible during the current pandemic.

This email has been sent through the NPG Manuscript Tracking System NY-610A-NPG&MTS

Confidentiality Statement:

This e-mail is confidential and subject to copyright. Any unauthorised use or disclosure of its contents is prohibited. If you have received this email in error please notify our Manuscript Tracking System Helpdesk team at http://platformsupport.nature.com .

Details of the confidentiality and pre-publicity policy may be found here http://www.nature.com/authors/policies/confidentiality.html

Privacy Policy | Update Profile

Disclaimer

This message (including any attachments) may contain confidential information intended for a specific individual and/or purpose. If you are not the intended recipient, please delete this message and notify the sender and the University immediately. Any disclosure, copying, or distribution of this message, or the taking of any action based on it, is prohibited as it may be unlawful.

In addition, the University specifically denies any responsibility for the accuracy or quality of information obtained through University E-mail Facilities. Any views and opinions expressed in the email(s) are those of the author(s), and do not necessarily represent the views and opinions of the University. The University accepts no liability whatsoever for any losses or damages that may be incurred or caused to any party as a result of the use of such information.
